# Genomic-to-space measurements reveal large-scale ocean nutrient stress

**DOI:** 10.1126/sciadv.aed8089

**Published:** 2026-06-05

**Authors:** Adam C. Martiny, Lucas J. Ustick, Toby K. Westberry, Michael J. Behrenfeld

**Affiliations:** ^1^ Department of Earth System Science, University of California, Irvine, Irvine, CA, USA.; ^2^ Department of Ecology and Evolutionary Biology, University of California, Irvine, Irvine, CA, USA.; ^3^ National Institute of Aquatic Resources, Technical University of Denmark, Lyngby, Denmark.; ^4^ Department of Botany and Plant Pathology, Oregon State University, Corvallis, OR, USA.

## Abstract

Phytoplankton growth and ocean primary production depend on a nutrient supply that fluctuates across seasonal to millennial timescales. Because surface nutrients and phytoplankton biomass recycle rapidly, they obscure the large-scale pattern of nutrient stress. Here, we integrate a satellite-derived index of phytoplankton physiology with hydrographic observations, omics biomarkers, and nutrient-addition experiments to understand the drivers of ocean nutrient stress. A clear biogeography emerges. Nutrient stress tracks nutricline depth and is stronger in nitrogen- than phosphate-limited waters, peaking where cells exploit rarer alternative nutrients. Seasonal variability dominates, but there are also clear signatures of major climate modes. Over the past two decades, surface warming has broadly intensified nutrient stress. A key exception is in southern hemisphere oligotrophic regions, where enhanced nitrogen fixation appears to offset stratification effects. This synthesis of hydrography, genomics, and satellite physiology exposes contemporary, climate-linked shifts in the large-scale distribution of phytoplankton nutrient stress.

## Teaser

Satellite, genomic, and hydrographic analyses reveal climate-driven shifts in phytoplankton nutrient stress across the ocean.

## INTRODUCTION

Over much of the global ocean, photosynthesis and growth of surface mixed-layer phytoplankton communities are limited by the availability of macro- or micronutrients (*1*). Most of these nutrients are delivered to the surface through vertical transport from deeper in the water column, the rate of which depends on the strength of upper ocean stratification. Water temperature is a dominant factor determining density gradients, so continued climate warming poses a major potential threat to ocean productivity due to a lower nutrient flux (*1*). Τhe upper ocean nutrient gradient (i.e., the nutricline) and associated flux rates have changed over the last 50 years, leading to a lower phosphate but not nitrate supply likely due to compensation from nitrogen-fixation (*2*). However, it is unknown how such hydrographic changes affect ocean phytoplankton physiology and growth.

Phytoplankton communities are complex adaptive systems (*3*) that adjust both uptake efficiencies and cellular requirements to ameliorate impacts of environmental change (*4*, *5*). Thus, it remains unknown how marine ecosystems respond to altered nutrient availability from changes in stratification (*6*). Insight into this question can be gleaned from nutrient-related patterns in phytoplankton physiology measured in the field (*7*). However, traditional bottle-based nutrient stress assessments are laborious, disrupt natural communities, and struggle to capture complex ecological responses. Omics-based measurements offer a noninvasive alternative. Recently, several studies, using the numerically dominant genus *Prochlorococcus*, identified gradients in nutrient stress type and severity (*8*–*10*). *Prochlorococcus* dominates tropical and subtropical open-ocean communities, and its genomic nutrient stress markers have been demonstrated to covary with mixed-community nutrient limitation (*8*). Thus, *Prochlorococcus* functions as a sentinel species reflecting broader community nutrient stress patterns. Yet, despite their advantages, such omics-based techniques are inherently limited to “snapshots” in space and time. To overcome this limitation, omics data are merged here with satellite observations to understand changes in phytoplankton nutritional status over the past two decades.

While satellite assessments of marine ecosystems and primary production often focus on surface chlorophyll concentrations alone, recent developments now provide additional information on physiological status through measured variability in cellular carbon-to-chlorophyll ratios (Θ_obs_) (*11*). Phytoplankton finely tune Θ_obs_ (on timescales of days) to precisely match light-harvesting capacity to energetic growth requirements dictated by local conditions (*12*). The light-dependent photoacclimation element (Θ_photo_) is a predictable function of daily median irradiance (*13*, *14*). Discrepancies between Θ_photo_ and Θ_obs_ reveal additional carbon-to-chlorophyll tuning in response to a reduction in growth rate. In laboratory experiments, as nutrient stress increases, cellular chlorophyll content is down-regulated in proportion to declining division rates and associated demand for energy (*13*). Anchored in this mechanistic understanding of phytoplankton biology, a nutrient stress metric, Θ′, is proposed as the ratio of Θ_photo_ to Θ_obs_. In chemostat experiments as well as theoretical analyses, Θ′ displays a clear relationship with growth rate changes associated with nutrient availability. Thus, Θ′ decreases as nutrient stress increases (see fig. S1 for a detailed analysis). Phytoplankton nutrient limitation is commonly defined by an increase in growth following nutrient addition, whether in controlled incubations or field experiments. Nutrient stress, in contrast, reflects the physiological, adaptive, and ecosystem-level costs of sustaining biomass production under suboptimal nutrient supply, expressed as a gap between realized and optimal growth conditions. Hence, nutrient limitation and stress may have distinct biogeographies (*7*). We hypothesize that the distribution of Θ′ predominantly reflects the large-scale suppression of phytoplankton growth by nutrient stress. Because Θ′ may also be modulated by temperature, species-specific regulation, and other factors, we first evaluate its relationship with diverse nutrient stress indicators before interpreting large-scale spatial patterns.

To understand variability in satellite-based Θ′, we used in situ nutrient measurements and genomic biomarkers from the Bio-GO-SHIP program (*8*, *15*). Rather than serving as a strict validation, these comparisons help elucidate if Θ′ reflects nutrient stress and the overall impact of large-scale nutrient supply (via the nutricline) versus element-specific stress (Fe, N, and P). We then use ∼20 years of Θ′ data from satellite remote sensing to quantify contemporary changes in nutrient stress. This analysis reveals spatial patterns linked to vertical nutrient transport and temporal trends suggesting clear sensitivity, as well as resilience, of phytoplankton communities to altered nutrient fluxes associated with ocean warming.

## RESULTS

### Drivers of Θ′ variability using field indicators of nutrient stress

**Fig. 1.**
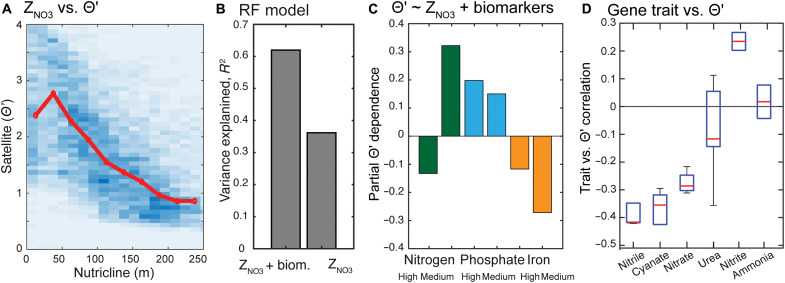
Environmental controls on Θ′ variability. (**A**) Significant correlation between the nutricline depth (Z_NO3_) and Θ′ (*R*_Pearson_ = −0.52, *P* < 1 × 10^−200^). Θ′ is the photo-acclimation normalized satellite-derived carbon-to-chlorophyll ratio (Θ_photo_:Θ_obs_). The nutricline depth is defined as the depth with 3 μM nitrate concentration. (**B**) Inclusion of genomic biomarkers nearly doubles the explained variability in remotely sensed Θ′ (left bar) beyond that captured by nutricline depth alone (right bar) in nonlinear (RF) models. (**C**) Integration of hydrography (i.e., nutricline depth) and genomic biomarkers shows a partial dependence of Θ′ on individual elemental stress type. Hence, Θ′ is lowest (most nutrient stress) under high N stress (left-most bar) or Fe stress (right two bars) and highest under P stress (blue bars) and medium N stress (second green bar). (**D**) Linear correlation between Θ′ and specific nitrogen utilization traits (table S1), showing that (from right to left) as phytoplankton switch to more complex nutrient sources under severe nutrient stress, Θ′ becomes increasingly low.

A comparison between satellite-derived Θ′ and field genomic, hydrographic, and phytoplankton physiology data revealed key insights into the environmental controls of Θ′ variability (Fig. 1). Rather than a direct validation of the in situ regulation of phytoplankton chlorophyll content, this analysis explores how Θ′ covaries with diverse indicators of nutrient stress. The expectation in oligotrophic regions is that Θ′ corresponds to surface nutrient availability but not nutrient concentration because the physiological state of phytoplankton depends on nutrient supply rates rather than standing stocks (*16*). Accordingly, we find insignificant correlations between Θ′ and nitrate and phosphate concentrations (fig. S2). Instead, we find that Θ′ varies systematically with the subsurface nutricline depth (Fig. 1A). As nutricline depth has been shown to closely and negatively correspond to the vertical nutrient flux in oligotrophic ecosystems (*2*), this correspondence (Fig. 1A) suggests that large-scale nutrient supply exerts a first-order control on Θ′. Specifically, Θ′ was elevated (>3) in regions with a shallow (or absent) nutricline and low (<1) in regions with nutriclines deeper than 150 m. We observe a positive correlation between Θ′ and in situ growth rate (fig. S3A) and a significant negative correlation between Θ′ and the change in growth rate following nutrient addition (i.e., the response to new nutrients is greatest in the most nutrient stressed populations) (fig. S3B) (*7*). Thus, Θ′ primarily reflects the phytoplankton growth penalty from nutrient stress and is closely tied to nutrient supply (as reflected by the nutricline). In contrast, Θ′ correlates weakly to sea-surface temperature (SST; fig. S2), indicating little direct impact by temperature. Last, the alignment between in situ and satellite nutrient stress indicators was strongest at a characteristic timescale of approximately 40 days (fig. S4). A peak correspondence at 40 days is consistent with the equilibration timescale of community-level physiological adjustments as well as larger sample averaging (*17*, *18*).

**Fig. 2.**
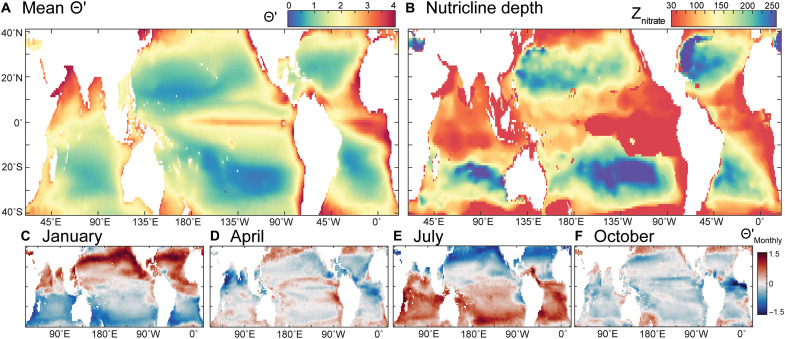
Climatological nutrient stress. (**A**) Mean nutrient stress based on remote sensing retrievals of Θ′. (**B**) Variation in the nutricline depth (Z_NO3_ = 3 μM) from World Ocean Atlas 2023. (**C** to **F**) Monthly Θ′ anomalies for January (C), April (D), July (E), and October (F). Figure S7 shows all monthly Θ′ anomalies. (A and C to F) Data are from MODIS-Aqua spanning July 2002 to December 2021 and averaged to 1° × 1° resolution. Θ′ is the phytoplankton photoacclimation-normalized carbon-to-chlorophyll ratio (Θ_photo_:Θ_obs_).

Despite the clear first-order dependence of Θ′ on nutricline depth (Fig. 1A), there is considerable unexplained variance in this relationship (Fig. 1B), indicating additional physiological and biogeochemical controls. For example, Θ′ was lower than nutricline-based expectations in equatorial regions (fig. S5), whereas it was higher than expected in the subtropical Northwest Pacific, most of the North Atlantic, and the entire Mediterranean Sea. We find that, in addition to nutricline depth, the inclusion of genomic biomarker observations in a nonlinear model helps explain a larger fraction of Θ′ variability (Fig. 1B). This finding indicates that Θ′ registers additional elemental stress signals (Fe, N, and P). For example, Θ′ is suppressed in equatorial regions where the nutricline is shallow and surface macronutrients are elevated (the signature of this condition appears in Fig. 1A as a “dip” in Θ′ at the shallowest nutricline depths). This suppression corresponds to regions with a high frequency of iron stress biomarkers. Θ′ was at its minimum in regions with high N stress and medium Fe stress (Fig. 1C). An example of this N/Fe stress condition with a low Θ′ is the South Pacific subtropical gyre (Fig. 2), which is generally considered the most oligotrophic ocean region. Nutrient addition experiments provide further support that high N stress and medium Fe stress regions result in the lowest Θ′ (fig. S3).

The biogeography of specific *Prochlorococcus* genome nutrient stress markers further reveals that traits associated with the use of rarer alternative nitrogen sources are among the most negatively correlated to Θ′ (Fig. 1D and table S1). Under nitrogen replete conditions, *Prochlorococcus* primarily assimilates ammonium. As reflected in both gene regulation controls (*19*) and biogeographic distribution (*8*), cells progressively switch to alternative forms when N stress rises. Here, we see that triple-bonded N compounds, including nitriles and cyanate, were the least attractive N sources (*20*, *21*). As seen in other studies, nitrate use by *Prochlorococcus* is also associated with severe nutrient stress (*22*). Urea is another alternative N compound, but we observe no correlation between Θ′ and the biogeography of urea utilization traits. This suggests a limited growth penalty for using urea, which aligns with *Prochlorococcus* achieving high in situ growth rates while primarily assimilating urea (*23*). The use of nitrite shows a positive correlation to Θ′, which is the opposite as predicted. However, *Prochlorococcus* primarily use nitrite in regions with a deeper mixed layer and presumably an elevated nutrient flux (*24*). Θ′ is relatively high under P stress conditions (Fig. 1C). This explains the relatively elevated Θ′ in the western Pacific, most of the North Atlantic, and the entire Mediterranean Sea (Fig. 2). All of these regions have a deep nutricline but are commonly known to display P stress (*16*). Even though cells overexpress many costly proteins including alkaline phosphatases, there appears to be limited growth depression under P stress (*25*). Hence, low nitrogen availability, whether being from a low vertical flux or a suppression of N fixation due to limited Fe supply, causes the lowest Θ′ and most severe nutrient stress conditions in the ocean.

Our comparisons between satellite observations and genomic, hydrographic, and physiological data indicate that variability in Θ′ primarily reflects nutrient stress, not only in terms of overall nutrient supply (as represented by the nutricline) but also registering specific forms of nutrient stress. Leveraging these findings and the spatiotemporal coverage advantage provided by satellite observations, we next use Θ′ data to evaluate nutrient stress in ocean biomes between 40°N to 40°S, which is the approximate habitat range of *Prochlorococcus* and encompasses two-thirds of the global ocean area.

### Biogeography of nutrient stress

The biogeography of Θ′ shows high nutrient stress in the subtropical gyres (Fig. 2A). Conversely, nutrient stress is low in upwelling and coastal zones, eastern boundary currents, and at higher latitudes with deeper mixing (Fig. 2A). Beyond this broad biogeography, differences are observed among the subtropical ocean gyres. For instance, phytoplankton in the highly nutrient stressed South Pacific subtropical gyre (*26*, *27*) are N or N/Fe stressed and exhibit the lowest values of Θ′ (Fig. 2A). In contrast, the North Atlantic is the most P stressed ocean region (*28*) and displays the highest Θ′ values among the subtropical gyres. Thus, Θ′ discrepancies between subtropical gyres align with shifts between N and P stress. In the broad equatorial Atlantic upwelling zone, Θ′ is high, suggesting negligible nutrient stress despite nitrate concentrations typically close to detection limits (fig. S5). In contrast, Θ′ is intermediate along the equator in the eastern and central Pacific and then intensifies again outwards of the upwelling source (Fig. 2A). In these areas of the Pacific, Fe is the most limiting resource for phytoplankton growth and residual surface nitrate is a persistent feature (fig. S5) (*29*). The overall biogeography of Θ′ closely follows spatial patterns in nutricline depth, but with more detailed gradients (Fig. 2, A and B). The differential effect of N versus P stress is manifested as generally lower Θ′ values in the southern (more N stressed) compared to the northern (more P stressed) hemisphere. Θ′ is highly correlated (*R*_Pearson_ = 0.98) across different satellite ocean color missions, evidencing the robustness of the observed signal (fig. S6). Together, these findings from remote sensing of Θ′ support a clear biogeography of nutrient stress.

### Temporal dynamics of nutrient stress

In addition to its climatological mean state (Fig. 2A), the severity of nutrient stress also exhibits strong temporal variability, with 59% of the variance in Θ′ following recurring seasonal shifts (Fig. 2, C to F, and figs. S7 to S12). Empirical orthogonal function (EOF) analysis associates the top temporal modes to frequencies of one or two annual cycles capturing the seasonal variance in Θ′ (figs. S8 and S9). Nutrient stress generally oscillates between being lower in winter and higher in summer for both hemispheres, thus following changes in stratification and likely nutrient supply changes. Tropical regions exhibit two Θ′ cycles per year, which corresponded to solar position and Θ′ maxima periods of elevated upwelling (*30*) (for additional site-specific examples, see fig. S13).

**Fig. 3.**
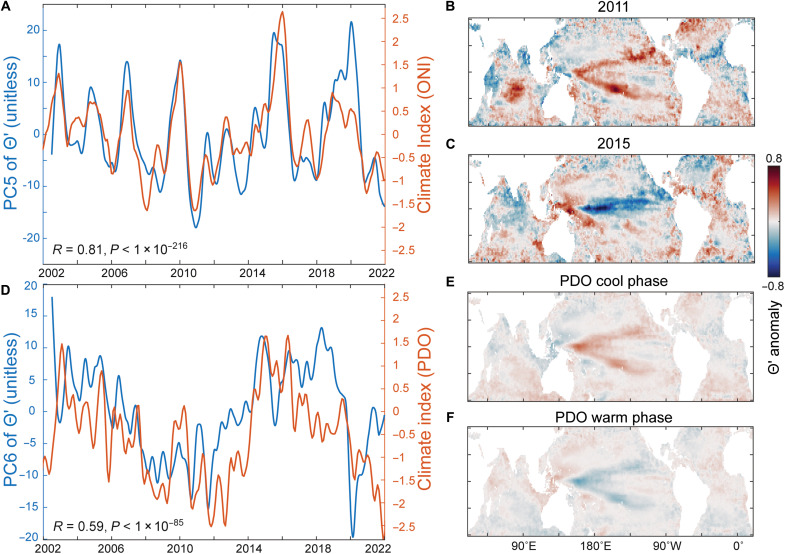
Link between climates cycle and ocean nutrient stress. (**A**) Comparison between the primary interannual nutrient stress mode of variability (PC5) and the Ocean Niño Index (ONI). (**B**) Anomaly in nutrient stress in 2011 illustrating a strong La Niña event. (**C**) Anomaly in nutrient stress in 2015 illustrating a strong El Niño event. (**D**) Comparison between the secondary interannual nutrient stress mode of variability (PC6) and the Pacific decadal oscillation index (PDO). (**E**) Mean annual anomaly during the PDO cool phase (years 2007–14 and years 2020–21). (**F**) Mean annual anomaly during the PDO during the warm phase (years 2002–06 and years 2015–19). The principal components (PC5 and PC6) are estimated from an EOF analysis of Θ′. The anomaly for all years is shown in fig. S14.

Superimposed on strong seasonal cycles, we also see interannual changes linked to both known climate cycles and, possibly, long-term anthropogenic trends. In a few regions, such as the equatorial Pacific upwelling zone, the majority of Θ′ variability is interannual (fig. S10). The top temporal mode with cycles longer than annual (PC5 from the EOF power spectra, fig. S12) has negative loadings in the equatorial Pacific Ocean, which switch to positive loadings in the western Pacific (fig. S12). There is a highly significant temporal correlation between PC5 and the ocean Niño index (ONI; Fig. 3A). This combination of spatial pattern and temporal mode support a link between PC5 and El Niño/Southern Oscillation (ENSO) climate variability. During the La Niña event in 2011, Θ′ was high at the edge of the upwelling zone due to increased upwelling, whereas the western Pacific Ocean expressed lower Θ′ (Fig. 3B). A similar pattern emerges during other La Niña events (fig. S14). In contrast, the strong El Niño event in 2015/2016 yielded depressed Θ′ along the edge of the equatorial upwelling zone and, again, showed an opposite response in the western Pacific (Fig. 3C). These findings illustrate the broad influence of ENSO cycles on the zonal intensity of nutrient stress in the equatorial Pacific Ocean and beyond. The next most important multiyear temporal mode (PC6) showed strong correspondence with the Pacific decadal oscillation (PDO; Fig. 3D). The cool PDO phase is characterized by increased temperatures in the western North Pacific, yielding a negative Θ′ anomaly (Fig. 3E). The opposite pattern is seen during the warm PDO phase (Fig. 3F). The Indian ocean dipole (IOD) causes a positive east-west temperature gradient (*31*). Strongly positive IOD events in 2007 and 2019/2020 led to decreased Θ′ on the western side of the Indian Ocean but depressed values on the eastern side (figs. S14 and S15). Thus, ENSO, PDO, and IOD natural climate cycle events all have clear associations with satellite Θ′ distributions and nutrient stress signatures.

### Long-term trends in ocean warming and nutrient stress

**Fig. 4.**
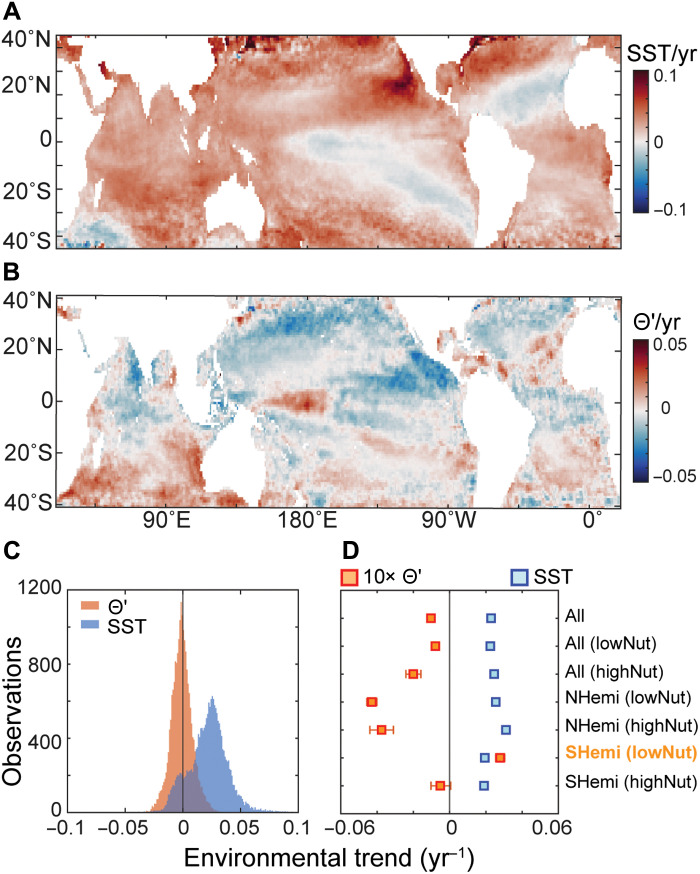
Contemporary trends in nutrient stress (Θ′) versus SST. (**A** and **B**) Spatial variation in long-term trends for (A) SST and (B) Θ′. (**C**) Frequency histograms for trends in Θ′ and SST from 2002 to end of 2021. The median trend for Θ′ is −1.3 × 10^−3^ year^−1^ and for SST is 2.3 × 10^−2^°C year^−1^. (**D**) Mean change in Θ′ versus SST by region. Changes in Θ′ are multiplied by 10 to keep on the same scale as SST. Nutrient conditions are defined by the depth of nitracline (using a 3 μM nitrate threshold) being above (highNut) or below (lowNut) 50 m. The 95% confidence intervals of the mean trend were calculated with 1 × 10^4^ bootstraps. The Southern Hemisphere, low nutrient region is highlighted (labeled in orange) to call attention to its opposite relationship to SST (i.e., less nutrient stress with warming) compared to the rest of the ocean. Annual anomalies for Θ′ and SST are shown in figs. S14 and S15, respectively.

Decadal-scale trends in upper ocean growth conditions were observed over the 2002–2021 satellite record. SST increased across 89% of the surface ocean area (Fig. 4A). The median rate of change in SST was 2.3 × 10^−2^°C/year, resulting in a 0.43°C average rise during the observation period (Fig. 4). Exceptions to this trend include regions of net cooling in the eastern North Atlantic, eastern South Pacific, and southwestern Indian Ocean (Fig. 4A). The long-term SST trends in the satellite record are modest compared to regional SST changes associated with the seasonal and climate cycles discussed above. Nevertheless, prolonged surface ocean heating is commonly expected to intensify water column stratification and, concomitantly, increase nutrient stress in phytoplankton.

Long-term trends in Θ′ exhibit greater spatial variability than SST, but the overall change is slightly (but significantly) negative (Fig. 4, B to D). Hence, similar to what we observed on seasonal and interannual timescales, warming is associated with more nutrient stress. However, the Θ′ trend displays higher variance, and there are hemispheric differences. In the northern hemisphere, Θ′ declined in most locations (Fig. 4). The main exception was the eastern North Atlantic Ocean, but this region with increasing Θ′ clearly coincided with ocean cooling. Thus, most northern hemisphere ecosystems conform to surface warming being paralleled by increased nutrient stress and vice versa. In the southern hemisphere, Θ′ declined with warming in high nutrient environments, but with a weaker response than seen to the north. In oligotrophic regions, Θ′ increased despite significant warming in large parts of the South Atlantic, Indian, and Pacific oceans. Thus, warming generally leads to increased nutrient stress, but there appears to be a secondary regulation causing less stress in southern hemisphere oligotrophic regions compared to the northern hemisphere. As discussed below, this observed spatial heterogeneity in nutrient stress responses to sustained SST change likely arises from a combination of physical and biogeochemical processes.

## DISCUSSION

Marine phytoplankton cellular chlorophyll content reflects a balance between energy acquisition and metabolic demand (*14*, *32*). In addition to light-driven photoacclimation, Laws and Bannister (*12*) provided an early demonstration of the strong influence of nutrient stress, reporting a consistent linear relationship between the chlorophyll:carbon ratio of a diatom and both nitrogen- and phosphorus-limited growth rate, a response later shown to be conserved across phytoplankton taxa (*32*). Such a shared response across taxa suggests a secondary role of biodiversity influencing Θ′. Although we lack global observations of phytoplankton diversity, phytoplankton communities within subtropical gyres are relatively stable in space and time, yet we observe substantial Θ′ variability, arguing against community composition as a primary driver of Θ′. Furthermore, we find little evidence for a major direct impact of temperature on Θ′ variability. Only few studies have experimentally tested how temperature affects phytoplankton chlorophyll content at a fixed growth rate (*33*, *34*). Here, the chlorophyll content declines predictably with increasing nutrient stress across temperatures. However, there was also a direct temperature impact on chlorophyll content, but with a magnitude much lower than seen in this study (*33*). In Eppley’s original work, a temperature impact on chlorophyll is also seen, but this effect disappears when cellular carbon-to-chlorophyll is normalized to growth rate (*34*). In our own study, we find that temperature explains less than 1% of Θ′ variance (fig. S2). On the basis of these considerations and the strong supporting evidence provided by our satellite Θ′ and field genomic, hydrographic, and phytoplankton physiology comparisons (Fig. 1), we propose that nutrient stress is the dominant factor controlling broad biogeographical and temporal patterns of Θ′ reported herein. Nevertheless, it should still be recognized that some of the smaller-scale spatial and temporal variability observed in Θ′ likely includes contributions from biodiversity (e.g., variations in accessory photosynthetic pigments), temperature changes (e.g., resulting in unbalanced growth), and local deviations from our global photoacclimation model.

Between 40°N and 40°S, open-ocean phytoplankton communities are numerically dominated by *Prochlorococcus*, which has a competitive advantage in terms of nutrient uptake when nutrients are extremely scarce because of its high surface area-to-volume ratio. In these ecosystems, nutrient and phytoplankton stocks alone poorly reflect physiological stress, which is instead captured through cellular acclimation and adaptation patterns (*35*). *Prochlorococcus* genomic biomarkers are particularly useful as indicators because their nutrient stress signatures covary with those of the broader phytoplankton community (*8*). The strong correspondence between *Prochlorococcus* stress biomarkers and satellite Θ′ thus reflects shared physiological responses across taxa. While transcriptomic data could, in principle, capture short-term responses, their coverage remains sparse, and results are highly variable. In contrast, metagenomic biomarkers of nutrient acquisition and assimilation offer a consistent, large-scale indicator of nutrient stress. Comparing these genomic patterns with Θ′ therefore serves not as a direct validation but as an integrative test of whether satellite-inferred physiological variability aligns with ecological adaptation to nutrient regimes. Collectively, the combination of hydrography, omics, and remote sensing data reveals a clear link between Θ′ and diverse nutrient stress markers.

Several physiological and ecological mechanisms explain why Θ′ indicates stronger stress under N than P stress. At the cellular level, the stoichiometric flexibility of P is greater than that of N, enabling cells to reduce P quotas when supply declines (*1*, *36*). Under P stress, phytoplankton can invest nitrogen into producing P-acquisition and recycling enzymes, but under N stress the limiting element is simultaneously required for synthesizing the uptake and photosynthetic machinery needed to overcome it. Consequently, the energetic and resource costs of responding to N stress might be proportionally higher. At the community and ecosystem scale, physiological constraints are compounded by differences in nutrient cycling rates. Dissolved organic P typically remineralizes faster than dissolved organic N (*37*), providing a more efficient P supply to the euphotic zone. Moreover, atmospheric deposition or diazotrophic N_2_-fixation introduce new N in the surface ocean, partially decoupling nutrient availability and vertical flux from depth (*2*). Hence, higher Θ′ in P stressed regions could also be due to this additional nutrient supply. Some combination of these cellular and ecosystem processes may thus yield the emergent pattern of lower Θ′ values under N and Fe stress relative to P stress observed in our analyses.

Examining the distribution and variability of phytoplankton physiological traits offers valuable insight into the overall physiological state of plankton communities and their environmental controls. This relationship is evident in monthly (Fig. 2), interannual (Fig. 3), and decadal (Fig. 4) shifts in Θ′. Both natural and anthropogenic warming of the surface ocean coincide with rising nutrient stress and suppressed phytoplankton growth. Yet, long-term satellite trends in Θ′ reveal a divergent pattern between hemispheres (Fig. 4), implying additional biogeochemical regulation. We propose that this divergence reflects contrasting nutrient regimes. Northern-hemisphere waters show greater phosphorus stress, indicated by low surface phosphate, deep phosphaclines, and presence of P-stress biomarkers (*16*). In contrast, southern-hemisphere regions experience broader iron-nitrogen stress. A reported 50-year decline in the phosphate-to-nitrate ratio in southern waters likely reflects enhanced stratification, reduced vertical supply, and compensatory increases in nitrogen fixation (*2*). Together, these dynamics suggest that warming amplifies nutrient stress through stratification, but that increased N fixation can partly offset this effect, reducing overall stress in the southern hemisphere. Where nitrogen fixation is already high, as in many northern regions, this buffering may not occur, and P stress persists. Thus, while ocean warming will undoubtedly affect marine ecosystems, the notion that “warming simply increases nutrient stress” is not supported by current satellite records (fig. S16). Instead, interactions among the N, P, and Fe cycles appear central. Sustained, global observations of phytoplankton physiology are therefore essential to refine our understanding of how climate change impacts ocean ecosystems.

## MATERIALS AND METHODS

### Data sources

Raw observations for this project have all been previously reported. Metagenomics samples (*n* = 990) are mainly from Bio-GO-SHIP (*15*). The intensity of nutrient stress is represented by the genomic index Ω*_XY_*, where *X* subscript refers to the specific form of nutrient stress (nitrate, phosphate, and iron) and *Y* refers to intensity (low, medium, and high). All Ω*_XY_* values are calculated as described previously (*8*). Surface nitrate and phosphate concentrations and nutricline depths (the depth horizon with [NO_3_^−^] = 3 μM) are based on bottle measurements from CTD casts and linear interpolation to find the 3 μM depth horizon, collocated on the same section cruises as the DNA samples or climatological means (Fig. 2). For a small subset of stations, the nitrate-based nutricline depth was estimated based on climatological data from World Ocean Atlas. It is worth noting that there is a very high correlation between nutricline depths calculated using different threshold values or using phosphate versus nitrate.

Satellite remote sensing observations used in the main manuscript are from the MODIS-Aqua sensor and represent the most recent mission reprocessing (R2022) (table S2). We also used comparable data products derived from SeaWiFS observations to relate results between sensors (see details below). Specifically, we used the standard distributed products of chlorophyll-a concentration (Chl, mg m^−3^), the particulate backscattering coefficient at 443 nm (b_bp_443, m^−1^), the backscattering spectral parameter (S_bp_, unitless), the diffuse attenuation coefficient at 490 nm (K_490_, m^−1^), daily incident broadband irradiance (PAR, mole quanta m^−2^ day^−1^), and SST (°C). Each of the products’ native spatial and temporal resolution and the time period of interest are given in table S2. The quantities b_bp_443 and S_bp_ were estimated using the generalized inherent optical property model (*38*) and converted to phytoplankton biomass (*39*). K_490_ was expanded to diffuse attenuation for PAR (K_PAR_) following Morel *et al.* (*40*).

Mixed layer depths (MLDs; m) were estimated from temperature and salinity profiles output by the data-assimilative HyCOM model (*41*) and a density threshold criterion of 0.03 kg m^−3^ (*42*). HyCOM output has the same spatial and temporal resolution as the MODIS-Aqua ocean color products (table S2). All ocean color-derived properties and MLD were down-scaled to 1° × 1° and 8-day resolution for the spatial and temporal mode analysis.

Monthly ONI and PDO indices were retrieved from the NOAA National Center for Environmental Information and based on ERSST version 5. The correlation coefficient and significance between EOF principal components and climate indices are based on a Pearson correlation on interpolated values (to match time points).

### Estimating Θ′

The symbol Θ has been historically used to denote the phytoplankton carbon to chlorophyll ratio (Θ = C:Chl), a quantity that registers the combined effects of light and nutrient availability on phytoplankton physiology (*12*, *43*). In this work, we define a new quantity, Θ′, which represents the component of C:Chl variability attributable to nutrient stress

$$\Theta^{\prime }=\frac{\Theta_{\text{photo}}}{\Theta_{\text{obs}}}$$ (1)

where Θ_obs_ is the satellite-observed C:Chl ratio and Θ_photo_ is the C:Chl variability due to photoacclimation to the median light level of the mixed layer. In other words, Θ′ is the satellite observed C:Chl relative to the photo-acclimation component of C:Chl. Here, Θ_obs_ was estimated directly from satellite retrievals of Chl and phytoplankton carbon biomass (C or C_phyto_) as described above and Θ_photo_ is estimated following a modified version of Behrenfeld *et al.* (*13*). The model of Behrenfeld *et al.* (*13*) integrates effects of diel variability in the underwater light field due to time of day and vertical mixing. This includes phytoplankton exposure to darkness during dawn/dusk and periods of vertical mixing that extend below the sunlit euphotic zone, which can affect cellular synthesis of Chl, and thus Θ. The model can be represented by a series of simple expressions that require only three globally available input quantities

$$\Theta_{\text{photo}}=\Theta_{\text{DM}}\times \Delta \Theta_{\text{SM}}$$ (2)

$${\Delta \Theta }_{\text{SM}}=\frac{1+e^{-0.15\text{PAR}}}{1+e^{-3I_{\text{ML}}}}$$ (3)

where Θ_photo_ is the combination of a baseline solution (Θ_DM_) corresponding to deep mixing conditions (MLD > 6 optical depths) and a “shallow mixing correction (ΔΘ_SM_)” for when the MLD is shallower than the deep-mixing criteria. In our application here, we have set Θ_DM_ to a constant value equivalent to the mean Θ_DM_ calculated over the entire MODIS-Aqua mission (=150 mgC mg Chl^−1^). PAR is the daily integrated broadband (400 to 700 nm) surface irradiance and K_PAR_ is the diffuse attenuation coefficient for PAR. The exponent in the shallow mixing correction contains the term *I*_ML_, which is defined as the median irradiance within the mixed layer (*11*)

$$I_{\text{ML}}=\text{PAR}\times e^{-K_{\text{PAR}}\times \text{MLD}/2}$$ (4)

Patterns of Θ_photo_ estimated from this model broadly match the behavior of other photo-physiological indices (e.g., the photosynthetic assimilation efficiency, cellular fluorescence, in situ Θ, etc.) and patterns in expected C:Chl (*13*). Θ_photo_ is not empirically fit to observed C:Chl data but computed mechanistically from light and mixing conditions, as previously benchmarked (*13*) and thus statistically independent from Θ_obs_.

### Satellite-based nutrient stress index

Here, we illustrate how the ratio of Θ_photo_ to Θ_obs_ yields a consistent, approximately linear relationship between Θ′ and a nutrient-induced suppression of growth rate. Chlorophyll content in phytoplankton cells is constantly adjusted in response to changes in growth irradiance and nutrient availability. For illustrative purposes, light-dependent cellular chlorophyll-to-carbon ratios (Chl:C) are shown in fig. S1 (blue symbols, heavy blue line) for the chlorophyte, *Dunaliella tertiolecta*, based on laboratory cellular chlorophyll concentrations (*44*), conversion of cell size to carbon concentration (*45*), and adjusted for a light-saturated, nutrient-limited division rate of 0.75 day^−1^. For these data, division rates (heavy red line in fig. S1A) remain light saturated down to ∼200 μmole photon m^−2^ s^−1^ and then begin to decrease because the rate of increase in Chl:C is insufficient to completely offset the effect of decreasing growth irradiance (*44*).

When nutrient availability changes for a given growth irradiance, phytoplankton adjust cellular Chl:C in direct proportion to their nutrient-regulated division rate (*12*, *32*). This response in Chl:C is depicted in fig. S1A by the four thin blue lines, where increases in nutrient availability relative to the baseline *D. tertiolecta* curve are represented by the two upper blue curves and decreases in nutrient availability are represented by the two lower blue curves (note that for these simulations, Chl:C is assumed to converge upon a single value as growth irradiance approaches zero because division rate becomes entirely light-limited at these lowest intensities and thus is independent of nutrient availability). Growth irradiance dependent division rates for the simulated range in nutrient availability are shown in fig. S1A by the red lines.

In our main manuscript, satellite observations of phytoplankton chlorophyll and carbon concentrations are used to evaluate large-scale patterns in nutrient stress. To isolate the nutrient-dependent signal from cellular chlorophyll changes associated with variable mixed layer light levels, we calculate a nutrient-stress index (Θ′) by comparing satellite-observed C:Chl (Θ_obs_) to a purely light-dependent photoacclimation element (Θ_photo_) from a model that predicts C:Chl for an average phytoplankton division rate based on median mixed layer light levels (*13*). The validity of this model has been supported by direct comparisons with field observations (*13*, *46*).

Figure S1A provides an opportunity to illustrate the basis for our Θ′ equation. Specifically, we assume that the heavy blue line in fig. S1A represents the baseline Chl:C relationship (Chl:C_photo_) against which we wish to evaluate the degree of stress in the other four nutrient regimes (Chl:C_obs_). A variety of expressions could be used for this stress index, but it is the simple Chl:C_obs_ to C_photo_:Chl ratio that yields an index linearly proportional to the degree of nutrient stress. This ratio is equivalent to the nutrient stress index employed in our main manuscript: Θ′ = Θ_photo_/Θ_obs_ (fig. S1B). If instead, we defined Θ′ as the ratio, Θ_obs_/Θ_photo_, the resultant index would exhibit an inverse and curvilinear relationship with degree of nutrient stress (gray symbols in fig. S1B), as would the normalized difference index, Θ′ = (Θ_obs_ − Θ_photo_)/Θ_photo_ (yellow symbols in fig. S1B).

We also examined how changes in Θ′ relate to chlorophyll concentration to evaluate whether Θ′ primarily reflects variations in chlorophyll content. Here, we observed a positive correlation of *R*^2^_Pearson,Monthly_ = 0.053 for monthly changes and *R*^2^_Pearson,Yearly_ = 0.025 for yearly changes. As expected, Θ′ is generally high in regions with elevated chlorophyll and vice versa (i.e., a positive correlation). This makes sense, as we expect high biomass in regions with low nutrient stress. However, changes in chlorophyll only explain a small proportion of the overall variance in Θ′.

### Characteristic timescale of biological responses to environmental change

We isolated remote sensing data that corresponded to the time and location of the metagenomic sampling and averaged Θ′ observations across different spatial and temporal scales. We averaged spatially at 1 × 1 pixels, 3 × 3 pixels, 5 × 5 pixels, 1° × 1°, 2° × 2°, and 5° × 5° degrees centered on the field observations, and temporally from ±2 to ±30 day in 2-day increments. We then generated random forest (RF) models to predict Θ′ from Ω_NH_, Ω_NM_, Ω_PH_, Ω_FeH_, Ω_FeM_, and nutricline depth for each time and space combination and calculated the total Θ′ variance explained. RF estimates were made in R version 4.2.1 (*47*) using the randomForest package version 4.7.1.1 (*48*). Each RF estimate was created with 500 trees (fig. S15).

We averaged the data spatially in a 2° × 2° grid and temporally across a 40-day window describing the characteristic timescale of match between physiological acclimation (Θ′) and adaptation (omics shifts). This averaging was selected due to its high *r*^2^ and correspondence to in situ measurements.

### Linking genomic and remote sensing of nutrient stress using nonlinear models

We created RF estimates for Θ′ averaged spatially in a 2° × 2° grid and temporally across a 40-day window using just nutricline and nutricline combined with genomic biomarkers (Ω_NH_, Ω_NM_, Ω_PH_, Ω_FeH_, and Ω_FeM_) as predictors. This modeling was done using the randomForest R package version 4.7.1.1 (*48*). The RF model was not used to predict satellite Θ′ but rather to quantify the relative nonlinear contributions of nutricline depth and nutrient-stress biomarkers to understand observed Θ′ variability at matched locations (fig. S17). Large-scale Θ′ distributions (Fig. 2) are derived directly from satellite observations, not RF predictions.

Gene abundances for trait correlations were calculated in the same manner as previously (*8*). Raw metagenomic sequences were filtered for quality and any adapter sequences were removed using Trimmomatic version 0.35 (*49*). For sequence recruitment, Bowtie2 version 2.2.7 (*50*) was used, mapping the reads to a reference database containing 115 genomes, which included representative strains from the main ecotypes of *Prochlorococcus*, as well as *Synechococcus*, *Pelagibacter*, and *Roseobacter*. This diverse selection was used to minimize the recruitment of closely related species. Bowtie2 was run with the following parameters: --no-unal --local -D 15 -R 2 -L 15 -N 1 --gbar 1 --mp 3. The resulting SAM files were then sorted and indexed into BAM files using Samtools version 1.3 (*51*).

The recruited reads were analyzed with Anvi’o version 5 (*52*). Open reading frames from the reference genomes were aligned and clustered through NCBI BLAST (*53*) and MCL (*54*). Gene clusters from the *Prochlorococcus* genomes were then extracted and classified into single-copy core genes (SCCGs) and genes within the flexible genome (non-SCCG). The sequence coverage for all non-SCCG genes was normalized by the average coverage of SCCG genes. This approach provides an estimate of the number of gene copies per individual genome within the sample. A *z*-score was then calculated for the normalized abundance of each gene. All genes were correlated to Θ′ averaged at 2° × 2° grid and temporally across a 40-day window and the linear correlation was calculated.

### Geospatial data analysis

EOF analysis was applied to identify spatiotemporal modes of variability in Θ′ using the “Climate Data Toolbox for Matlab” (CDT) (*55*). A long-term linear trend was first removed. The power spectral density was estimated using the Matlab function “periodogram” embedded in the CDT function “plotpsd.” Matlab function “smooth” with a “Local regression using weighted linear least squares and a first-degree polynomial model (lowess)” filter with a window size of 20 was applied to visualize temporal trends. We applied a variance model for each spatial grid point (*n* = 20,822) with yearly (*n* = 20) and monthly (*n* = 12) factors using Matlab “anovan.” Jointly estimated variances attributed to either yearly or seasonal changes were calculated as the sum-of-squares for each factor divided by the total sum of squares.

### SeaWiFS comparison

Satellite data from the SeaWiFS mission were used to corroborate patterns reported for MODIS-Aqua (fig. S6). To remove systematic biases between the two missions, we extracted overlapping years (2003–2007) and evaluated mean differences in the basic properties of Chl and b_bp_443. We corrected SeaWiFS Chl by subtracting 0.012 mg m^−3^ and SeaWiFS b_bp_443 by adding 1.71 × 10^−5^ m^−1^. *R*_pearson_ = 0.74 for all data points and *R*_pearson_ = 0.97 for the mean values during the overlapping period.

## Supplementary Materials

### This PDF file includes:

Supplementary Text Figs. S1 to S17 Tables S1 and S2 References
