# Supplementary material for: Genomic-to-space measurements reveal large-scale ocean nutrient stress
Source: Sci Adv. 2026 Jun 5;12(23):eaed8089. doi: 10.1126/sciadv.aed8089 (PMC13240183; doi:10.1126/sciadv.aed8089)
Supplement: Supplementary file 1 — Supplementary Text Figs. S1 to S17 Tables S1 and S2 References [file sciadv.aed8089_sm.pdf]

Science Advances
AAAS

Supplementary Materials for

Genomic-to-space measurements reveal large-scale ocean nutrient stress

Adam C. Martiny *et al.*

Corresponding author: Adam C. Martiny, amartiny@uci.edu; Michael J. Behrenfeld, mjb@oregonstate.edu

Sci. Adv. **12**, eaed8089 (2026)
DOI: 10.1126/sciadv.aed8089

This PDF file includes:

Supplementary Text
Figs. S1 to S17
Tables S1 and S2
References

Supplementary Text

Additional methodological considerations. There are several methodological considerations related to how we estimate nutrient stress. First, we initially validated *Θ'* using *Prochlorococcus* biomarkers. *Prochlorococcus* is the most abundant phytoplankton lineage between 40˚S and 40˚N but rare at higher latitudes (*56*). This biogeography may explain the poor correspondence between remotely sensed *Θ'* and *in situ* metrics of nutrient stress in coastal and polar biomes. As a result, we restricted the analysis to the subtropical and tropical ocean. The low latitudes also include a wide diversity of other phytoplankton lineages, but the difference in the nutritional status among specific taxa are less understood (*57*). However, past comparisons between genomics biomarkers, whole community bottle experiments, and biogeochemical models showed high correspondence (*8*). As noted in the main manuscript, *Prochlorococcus* has the smallest cell size and associated highest affinity for nutrient uptake among common marine phytoplankton, suggesting that other lineages likely experience growth restrictions when *Prochlorococcus* is nutrient stressed (*58*).

Second, laboratory and field experiments suggest that light acclimation and nutrient growth restrictions are the main controls on phytoplankton C:Chl regulation (*12*). However, other factors, including the presence of non-functional photosystems under iron stress (*59*), shifts in community composition (*60*), and temperature may influence this ratio but with a smaller impact.

Third, the characteristic timescale of change in mixed layer growth conditions relative to phytoplankton growth responses is important in determining whether or not the observed *Θ'* represents a steady-state solution (*61*). Phytoplankton adjust cellular chlorophyll levels on the order of days. However, the comparison between genomic and satellite observations suggests an alignment on the order of ~3 weeks. We interpret this longer period of alignment as reflecting the pace of turnover in allele frequencies and thus an eco-evolutionary response, which may be slower than cell physiology. The detected biological response timescale aligns with various microbial time-series observations (*62*, *63*).

Fourth, the EOF analyses indicated that the top eight modes of variability explained 60% of the total variation in *Θ'*. In comparison, the top eight modes of SST explain 89.7% of the variance. We attribute this difference to the fact that SST is a singular remote sensing product, whereas *Θ'* is derived from a combination of five products. The incorporation of multiple products likely introduces additional uncertainty. However, the variance-explained ratio of long-term to seasonal modes was one to four for both SST and *Θ'*. Thus, ocean climate cycles affected both signals in equal proportions.

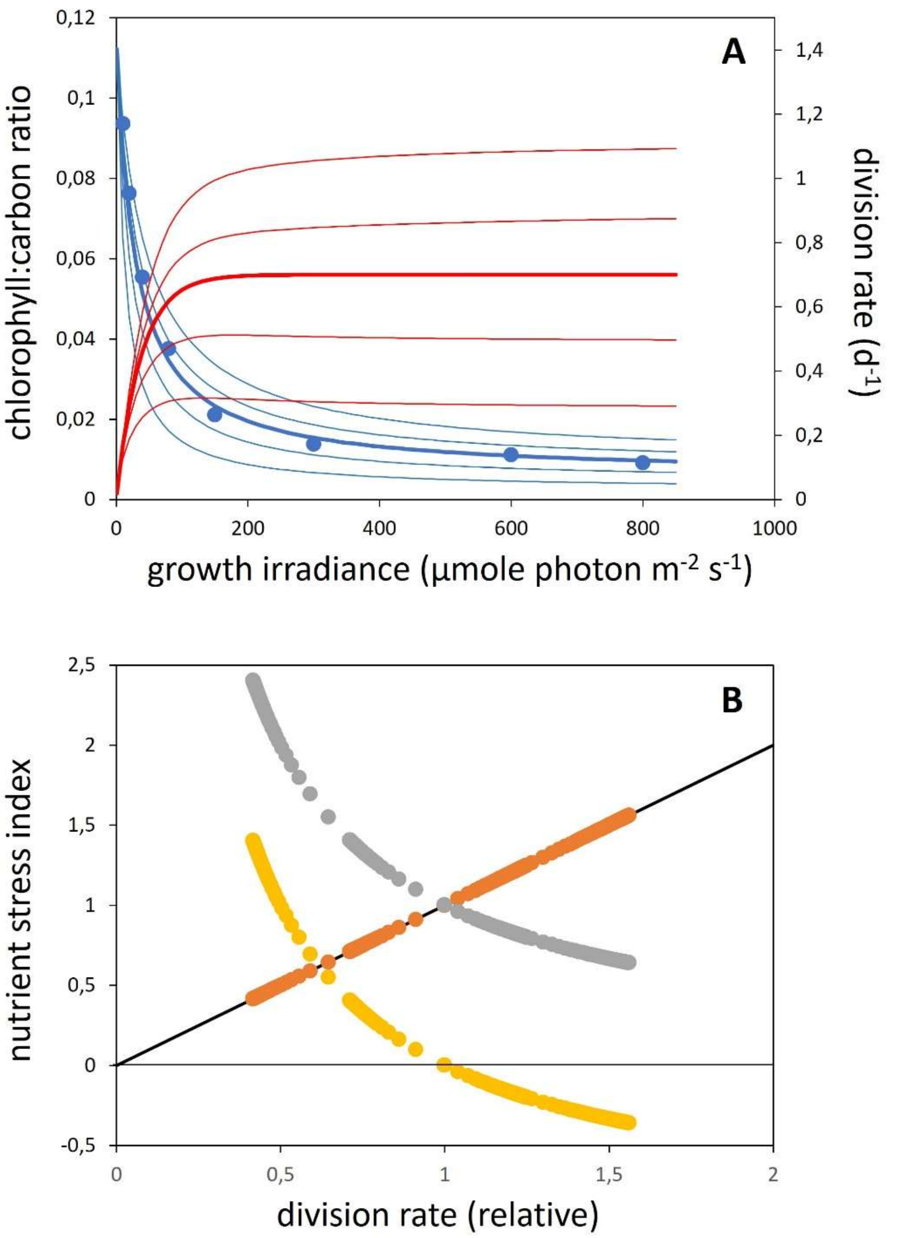

Figure S1. Photoacclimation responses in phytoplankton and selection of a nutrient stress index. (**A**) Representative relationships between phytoplankton Chl:C ratios and growth irradiance for a range in nutrient availability (blue lines) and associated light-dependent cell division rates (red lines) (see Methods). (**B**) Alternative expressions for a nutrient stress index (Θ') describing the degree of nutrient limitation relative to the baseline condition represented by the baseline relationships depicted in panel a by the heavy lines. Orange symbols: $\Theta' = \Theta_{photo}/\Theta_{obs}$. Gray symbols: $\Theta' = \Theta_{obs}/\Theta_{photo}$. Yellow symbols: $\Theta' = (\Theta_{obs} - \Theta_{photo})/\Theta_{photo}$. Black line indicates the 1:1 relationship.

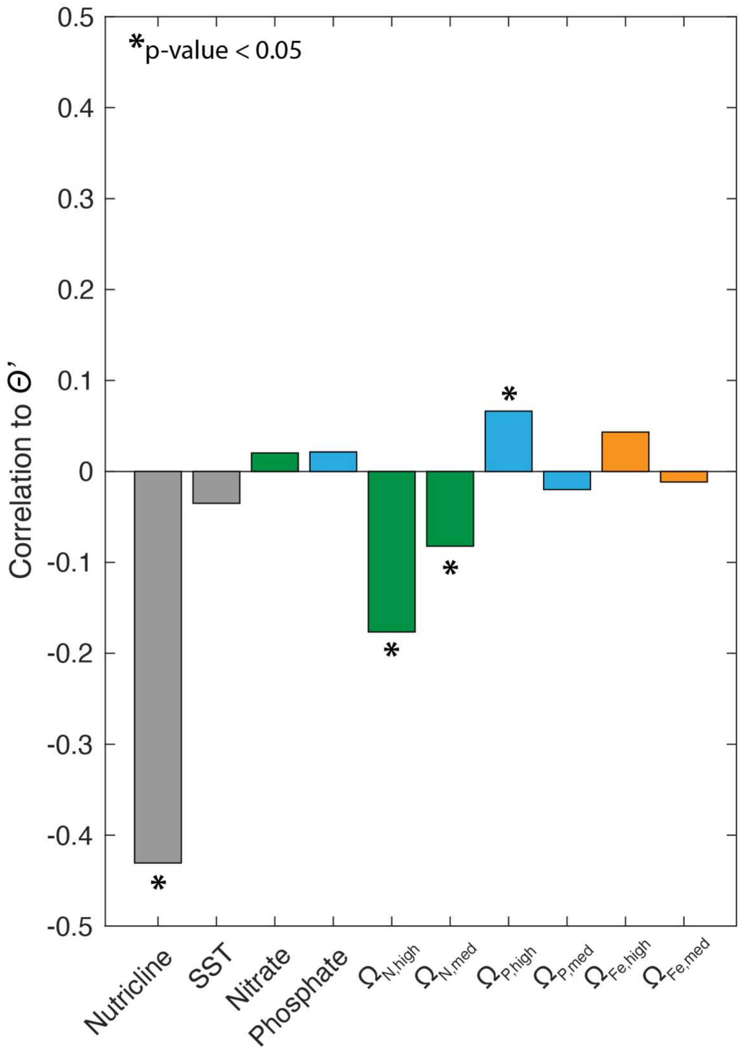

Figure S2. Comparison of *in situ* measurements and nutrient stress (*Θ'*). Pearson correlation between individual *in situ* hydrographic and genomic observations and *Θ'*. * denotes significant correlations (p value < 0.05). The best predictor is nutricline depth (the depth horizon with 3 μM nitrate). The comparison is done only with sites where we have genomic biomarker data (n = 1137). Hence, the correlation to nutricline depth is a little different from Fig. 1, where we compared the global variation in *Θ'* and nutricline depth using all grid boxes.

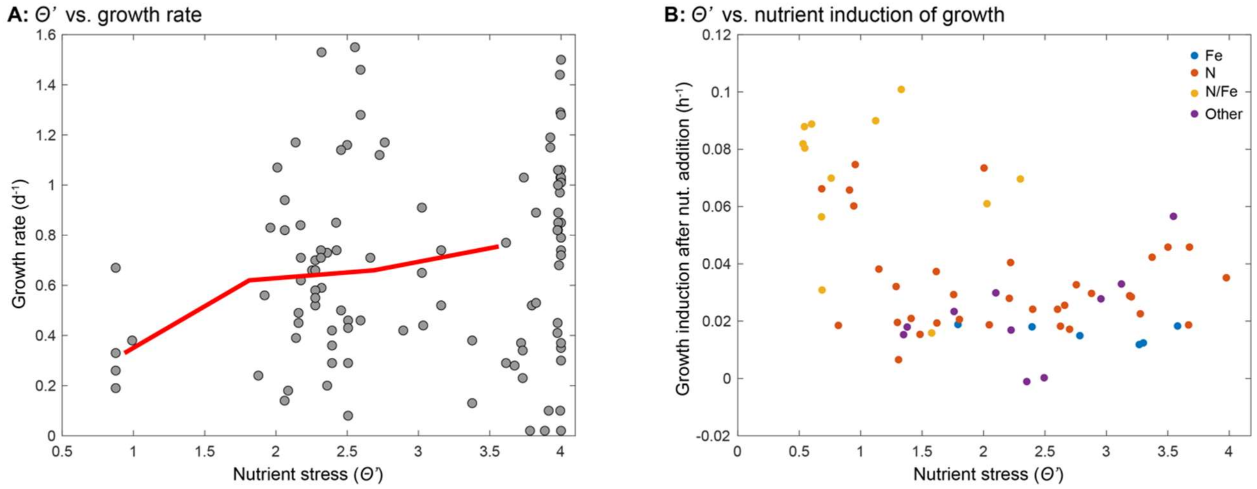

Figure S3. Correspondence between satellite observations of nutrient stress (*Θ’*) and phytoplankton growth rates (**A)** Positive relationship between *Θ’* and phytoplankton growth rate ($R_{spearman}$ = 0.20, $p < 0.05$). Here, growth rate is estimated using deck-board dilution experiments with no nutrient additions (n = 104)(*64*). Growth rate is normalized for temperature using a Q_{10} =1.88. The red line represents a running median. (**B)** Phytoplankton growth is most strongly induced following nutrient additions at low *Θ’* ($R_{spearman}$ = -0.31, $p < 0.005$). Growth induction is based on bottle experiments where combinations of nutrients are added. This confirms that *Θ’* is lowest under N/Fe and N limitation. Growth is estimated as the log-change in chlorophyll between the initial (t = 0 h) and final time point as described previously (*7*). Here, we compare mean *Θ’* from the source water location to the nutrient addition (as labelled) with the maximal growth induction.

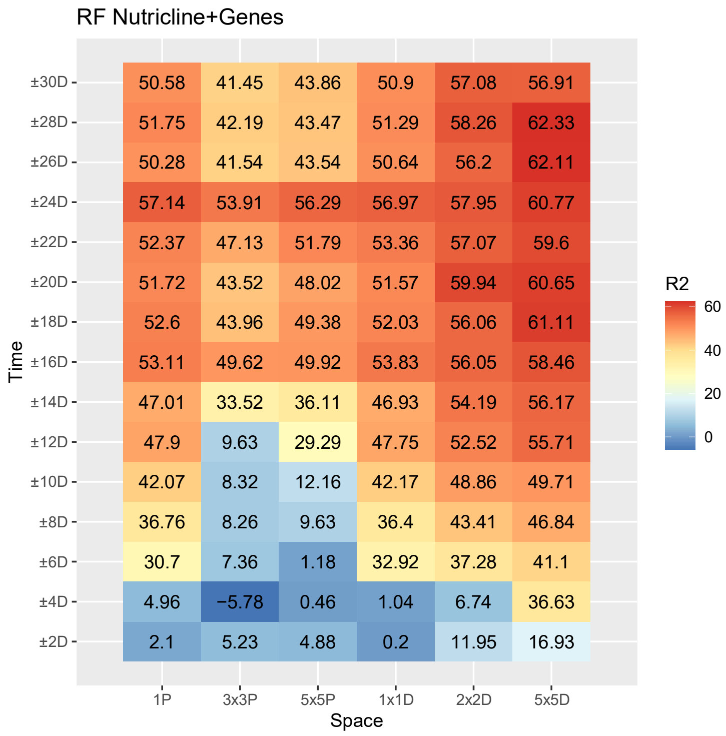

Figure S4. Spatial and temporal variation in match-up between *in situ* and satellite remote sensing observations (*Θ'*) of nutrient stress. We binned satellite observations of *Θ'* progressively at increasing spatial (going from one 1/12˚ pixel to 5˚-by-5˚ bins) and temporal (5-days to 2 months) scales surrounding the in situ genomic observations. The correspondence was partially dependent on the bin size which was likely due to a reduced standard error of having more observations. However, we also observed high correspondence at 2˚ and ~1 month bins suggesting that the in situ observations represented an integration of *Θ'* at this spatio-temporal scale.

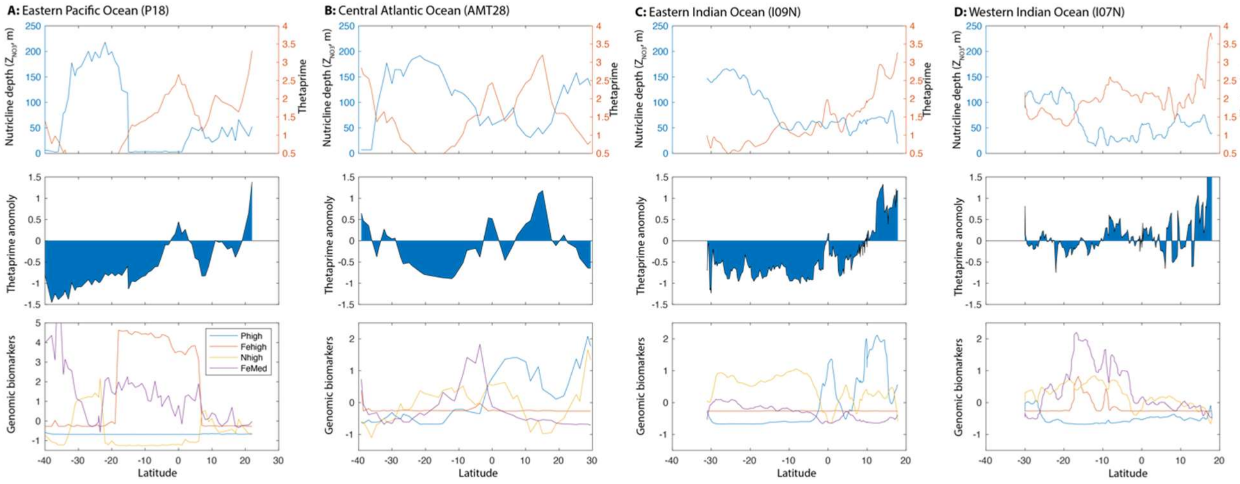

Figure S5. Correspondence between nutrient stress measured using *in situ* vs. remote sensing observations across ocean basin transects. Direct comparison of *in situ* estimated and remote sensing nutrient stress across the (**A**) Pacific, (**B**) Atlantic, (**C, D**) and Indian Ocean cruise sections as well as observed nutricline depth, *Θ'* anomaly, and genomic biomarkers. *Θ'* anomaly is calculated as the difference between observed *Θ'* and *Θ'* estimated using a linear fit to nutriclines (Fig. 1). Thus, the anomaly represents when *Θ'* deviates from expected from nutricline information alone. Areas with negative anomalies generally correspond to regions with N or Fe stress (based on genomic biomarkers), whereas areas with positive anomalies correspond to regions with P stress. This is particularly clear with the Eastern Indian Ocean transect (panel **C**), where the nutricline is at constant depth north of 15˚S but *Θ'* varies in tandem with shifts in N versus P stress.

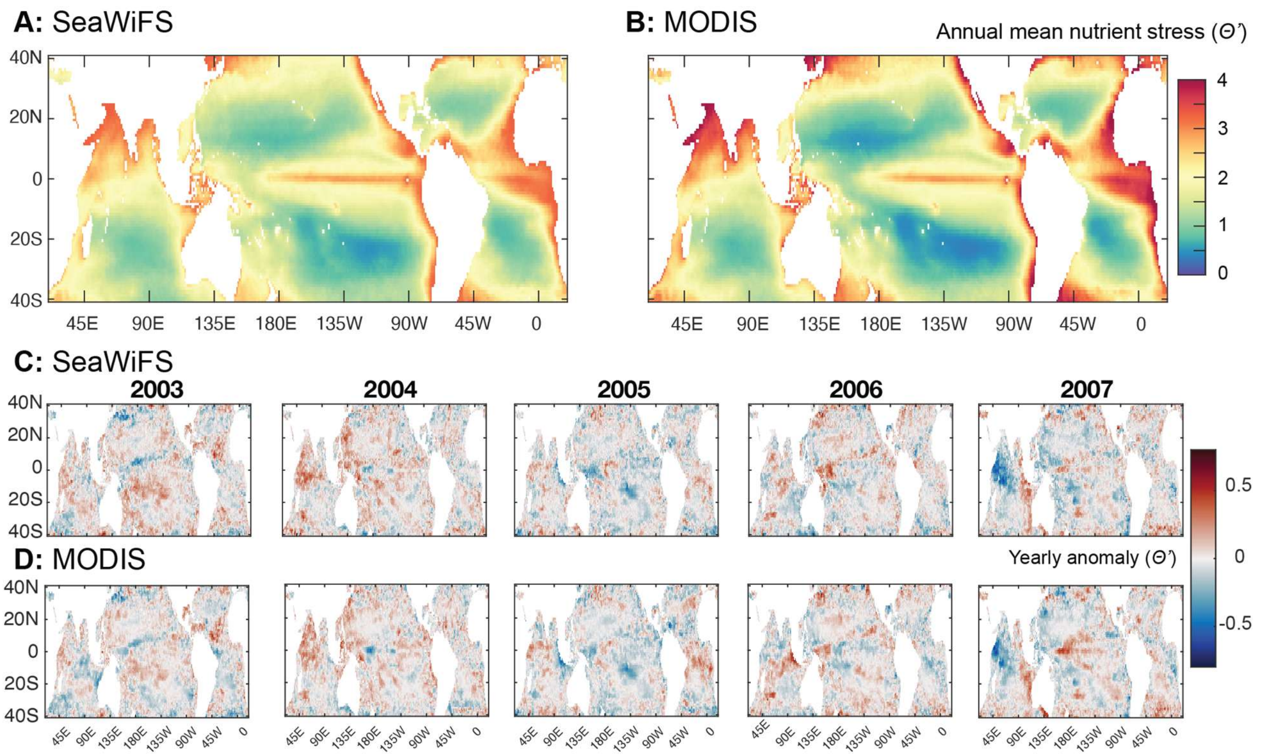

Figure S6. Comparison of global mean nutrient stress measured with different satellite sensors. Global mean nutrient stress for (**A**) SeaWiFS and (**B**) MODIS. Parallel annual changes in nutrient stress for (**C**) SeaWiFS and (**D**) MODIS. The data represent the overlapping period between 2003 and 2007. The Pearson correlations between measurements from both missions were 0.98 for global mean values and 0.75 for all individual data points. The panels show high correspondence between both global means and annual deviations between the two satellite ocean color missions.

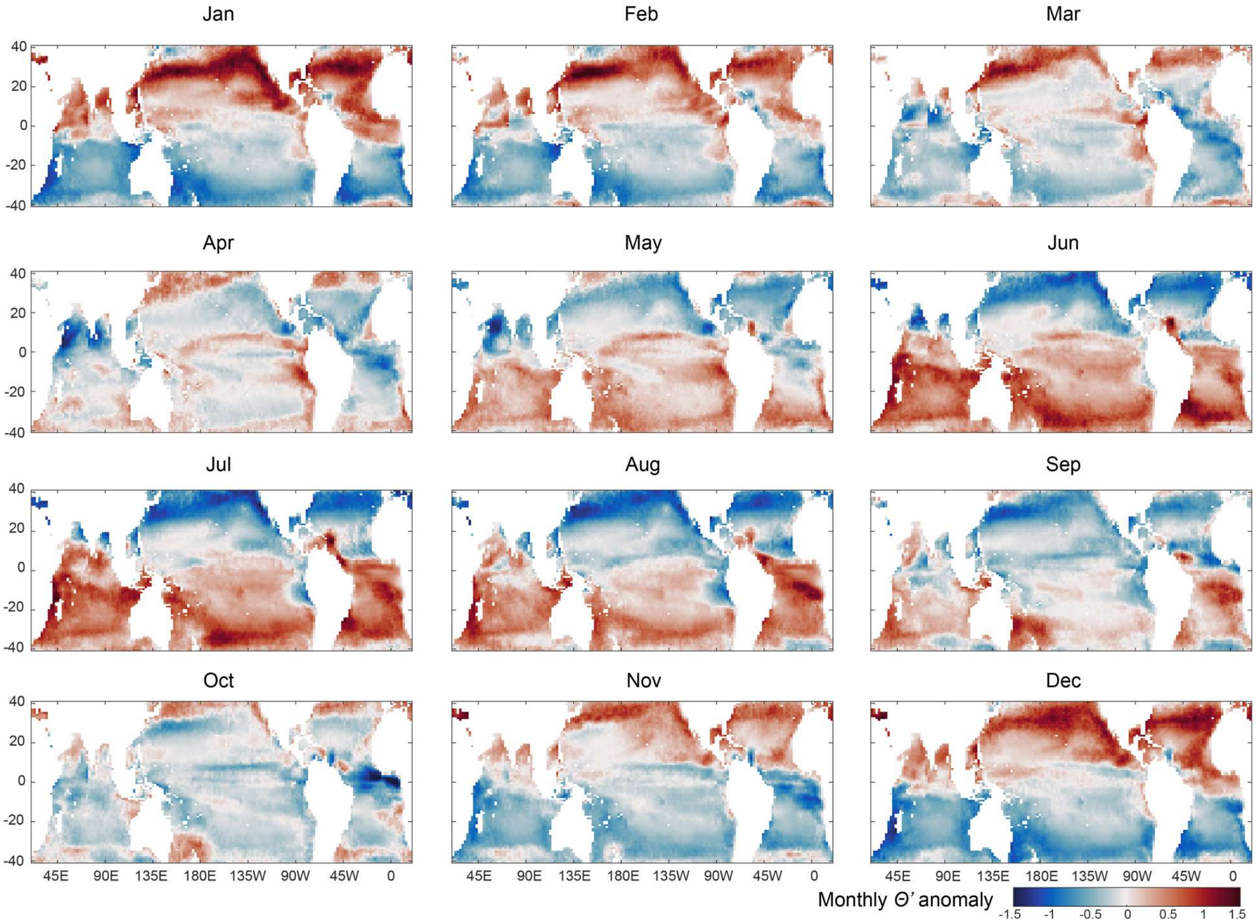

Figure S7. Monthly anomalies in nutrient stress, illustrating clear monthly shifts in *Θ'*. Over the annual cycle, nutrient stress is highest (and *Θ'* lowest) in the southern hemisphere during January, February and March. In March, April and May, nutrient stress increases in equatorial regions, particularly in the tropical Atlantic Ocean. Nutrient stress increases in the northern hemisphere starting in May and remains elevated until September. In October and November, nutrient stress is again high in tropical regions. Finally, elevated nutrient stress once again appears in the southern hemisphere beginning in November.

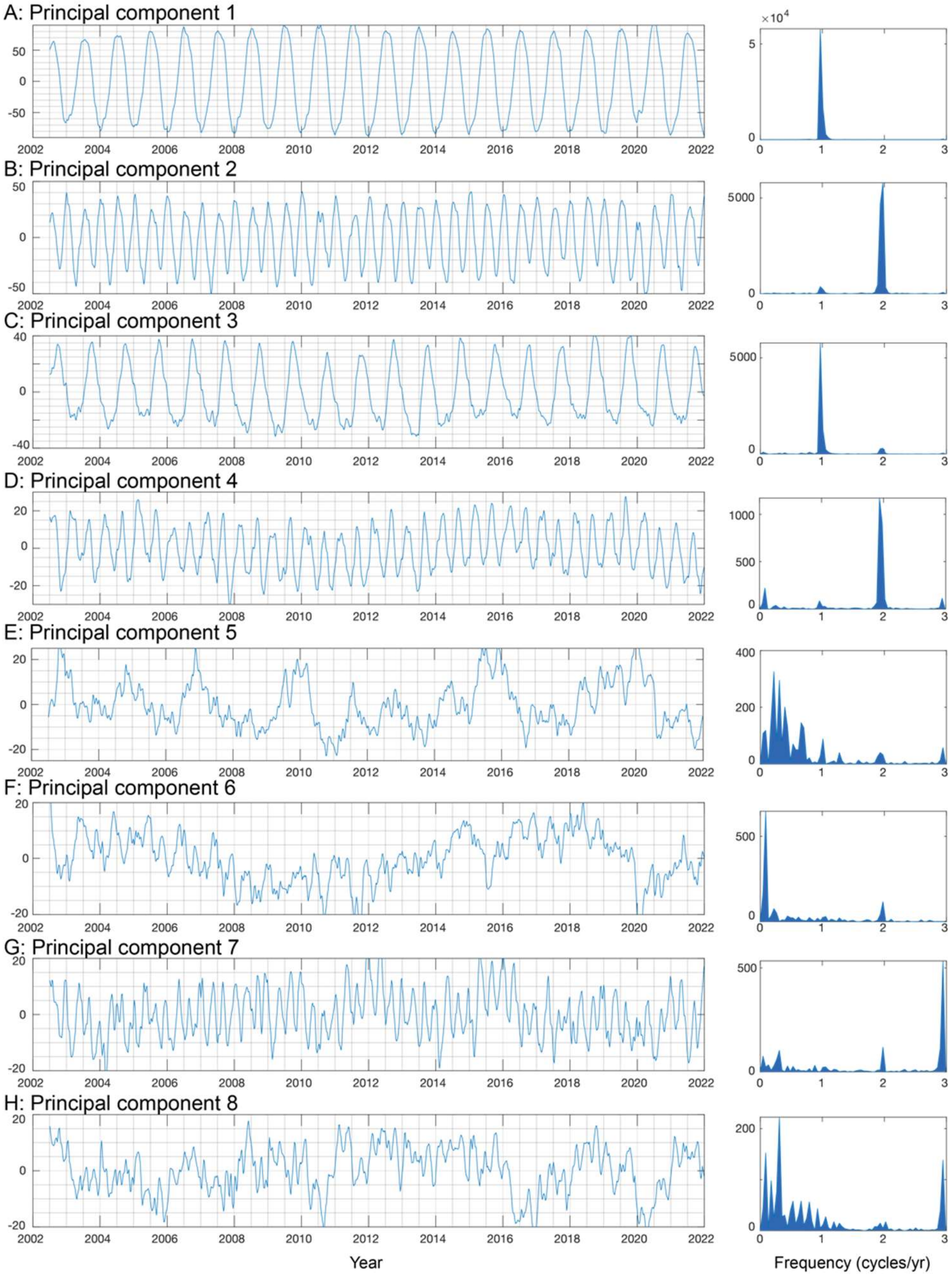

Figure S8. Contemporary modes of nutrient stress. (**A-H**) The temporal dynamics of the first eight principal components (left column) and their associated power spectra (right column) derived from an empirical orthogonal function (EOF) analysis. PC1 – PC4 captures most of the seasonal variation, whereas PC5 – PC8 are associated with variation at longer timescales. The nutrient stress data (*Θ'*) was detrended prior to the EOF analysis.

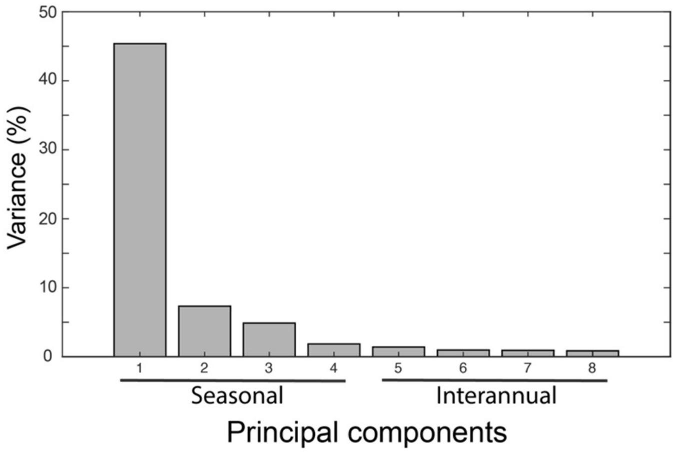

Figure S9 Variance associated with different modes of variations. The nutrient stress data (*Θ'*) were detrended and then subject to an EOF analysis. PC1 – PC4 are mainly seasonal and, combined, explain 59.4% of total variance. PC5 – PC8 mainly reflect long-term variation and summed explain 4.2% of the observed variation.

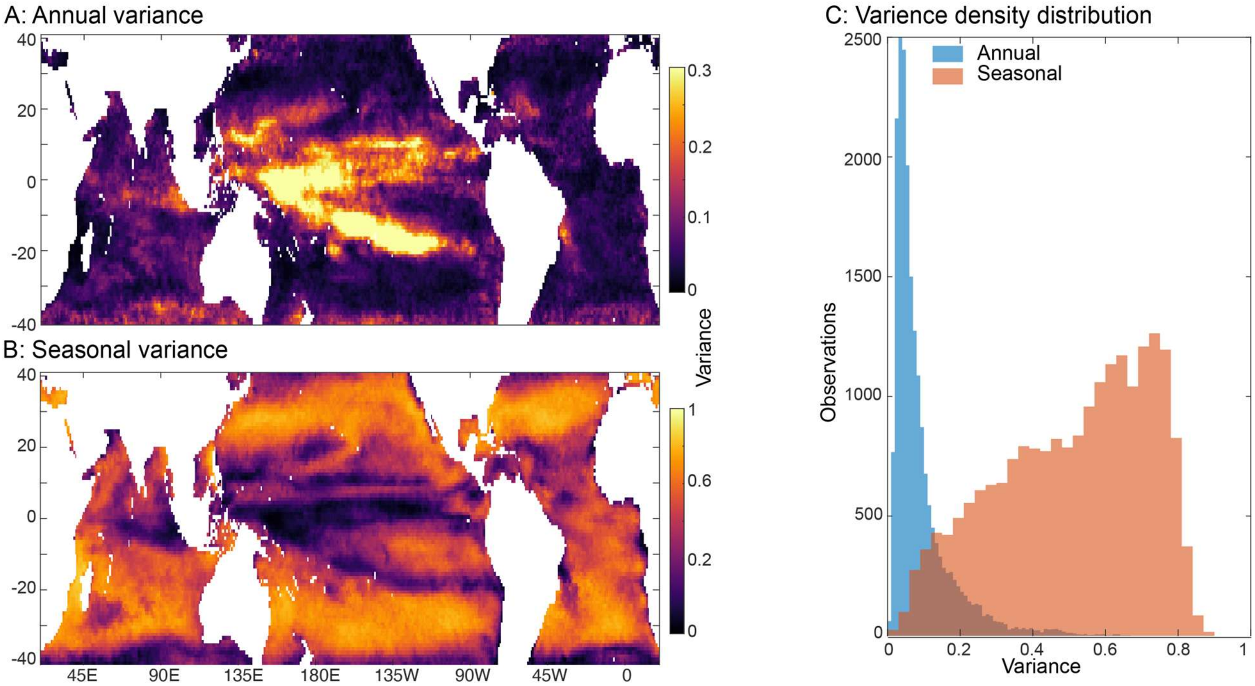

Figure S10. Temporal partitioning of variance in nutrient stress. Variance associated with (**A**) interannual and (**B**) seasonal changes in *Θ'*. (**C**) Histogram of variance associated with annual and seasonal changes in *Θ'*. The highest interannual changes are observed at the edge of the equatorial Pacific Ocean upwelling zone but lower in most other regions. Many regions show strong seasonal variance although this pattern is not strictly latitudinal. As such, many tropical regions also show strong seasonality in nutrient stress.

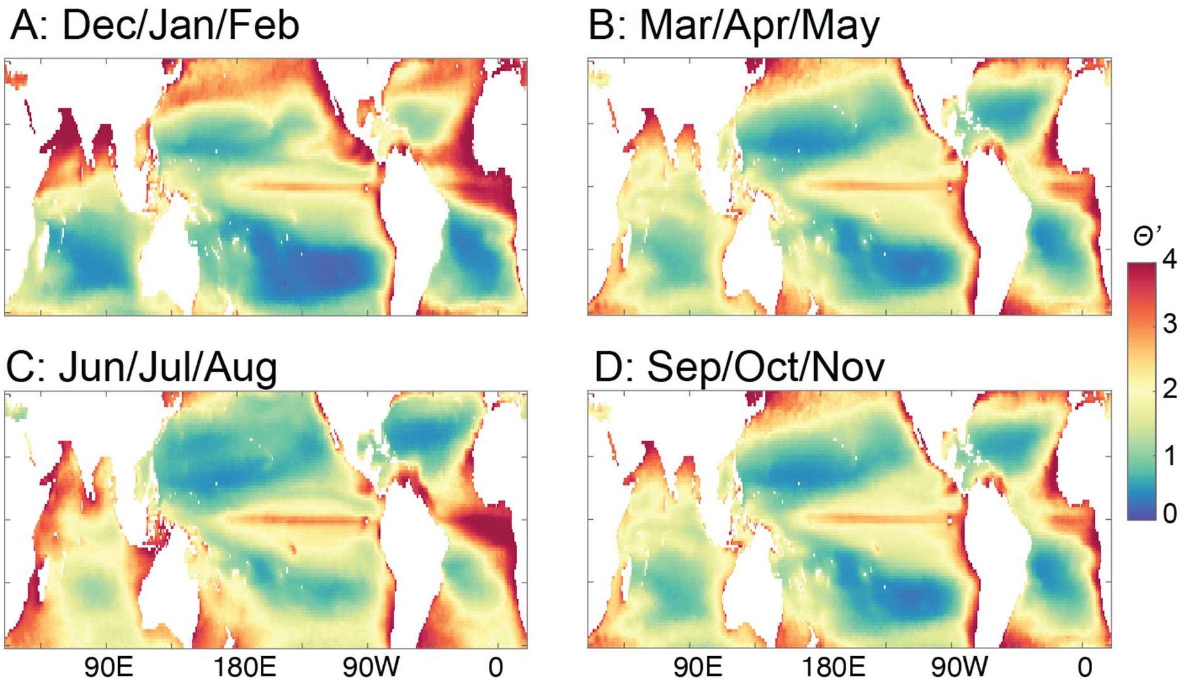

Figure S11: Seasonal mean nutrient stress (*Θ'*) for boreal (**A**) winter (December, January, and February), (**B**) spring (March, April, May), (**C**) summer (June, July, and August), and (**D**) fall (September, October, and November).

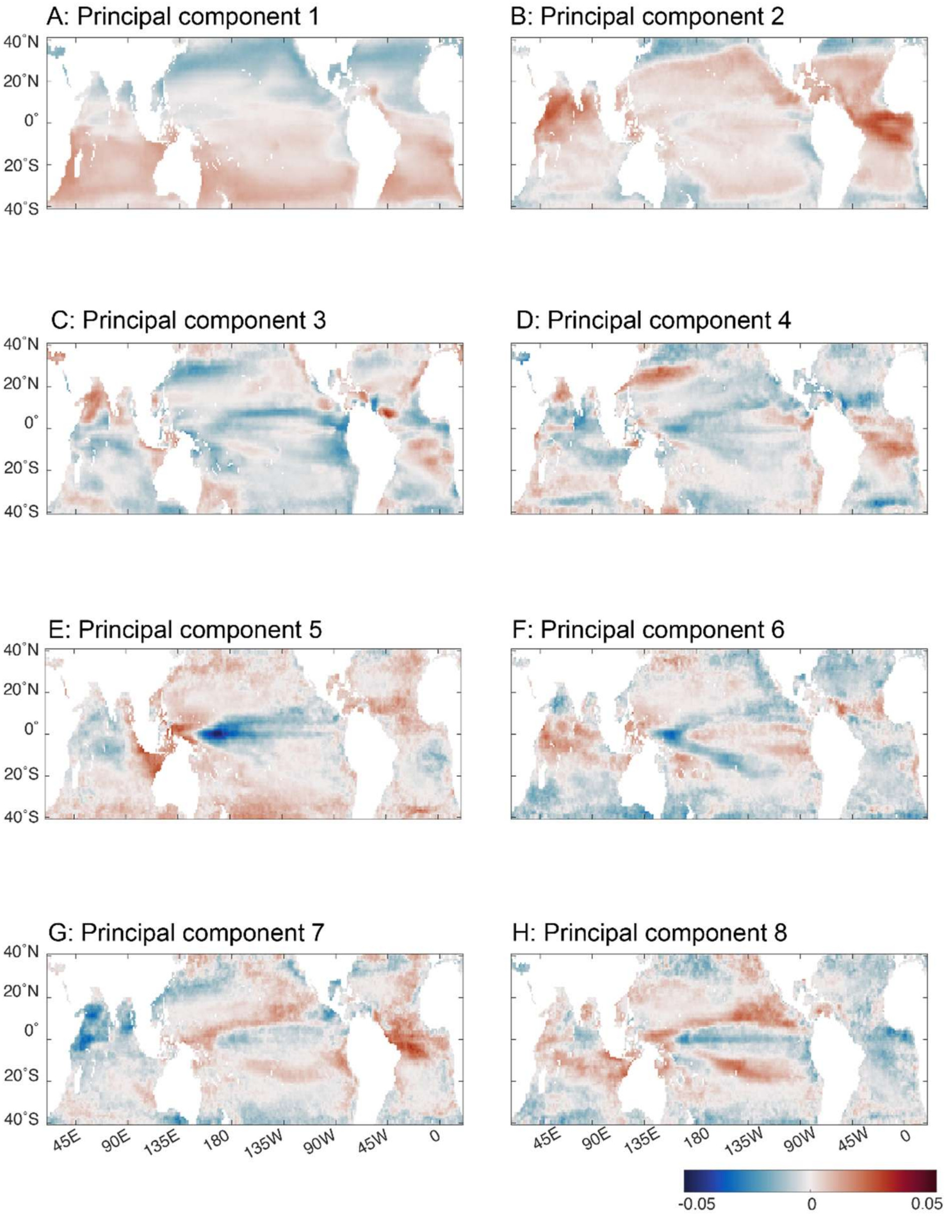

Figure S12. Global variation in temporal modes of nutrient stress. Panels **A – H** show the spatial loadings of each of the top eight principal components. The temporal modes are estimated using empirical orthogonal functions (EOF) and PC1-8 represent 63.6% of the total variance. PC1 captures most of the annual oscillation in nutrient stress and the loadings clearly illustrate the opposing cycle between hemispheres. PC2 captures most the variation in tropical regions with peaks separated by 6 months (2 cycles yr^{-1}). PC5 represents the primary inter-annual mode of variation and is strongly correlated to ENSO cycles (see Fig. 3 in main manuscript). The spatial loading pattern confirms this connection with strong opposite zonal loadings in the tropical Pacific Ocean. PC6 represents the secondary multi-annual mode of variation and is strongly correlated to PDO cycles.

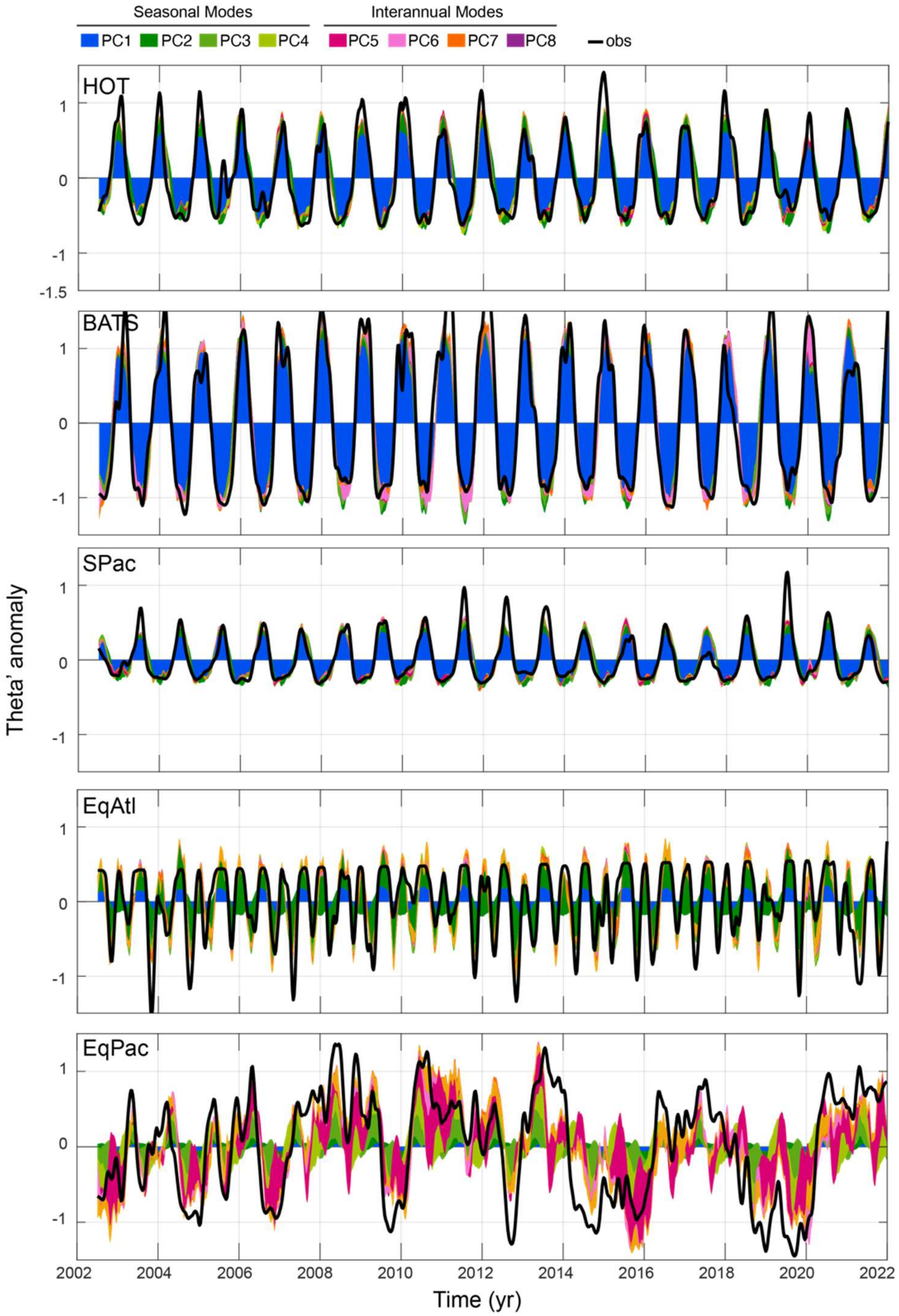

Figure S13. Temporal modes of variability in nutrient stress at specific ocean sites. Stations: HOT (23˚N, 158˚W) in the North Pacific subtropical gyre, BATS (32˚N, 64˚W) at the northern edge of western North Atlantic subtropical gyre, SPac (25˚S, 130˚W) in the eastern South Pacific subtropical gyre, EqAtl (0˚, 10˚W) in equatorial Atlantic Ocean, and EqPac (1˚N, 170˚E) in the equatorial Pacific Ocean. The colors represent the contribution from the top 8 principal components from an EOF analysis. Three stations (HOT, BATS, and SPac) were located within separate subtropical gyres. All showed distinct seasonal cycles predominantly with peaks in the summer. However, the amplitude varied with the lowest at HOT and the highest in the center of the South Pacific subtropical gyre. There was also a strong seasonal cycle in nutrient stress in the equatorial Atlantic Ocean. However, this station (and most of the tropical Atlantic and Indian Ocean) had two annual cycles matching shifts in radiative forcing and stratification. Here, the Θ' minima corresponded to periods with elevated upwelling likely relieving nutrient stress. The EqPac station is located at the edge of the Pacific Ocean equatorial upwelling zone. This station displayed a

very distinct temporal pattern with limited seasonal oscillations and strong interannual shifts. At EqPac, there were also several large spikes in Θ' not captured by the major principal components, suggesting additional episodic events. In sum, most variability in nutrient stress was associated with seasonal cycles, although some regions experienced significant variability at other temporal scales.

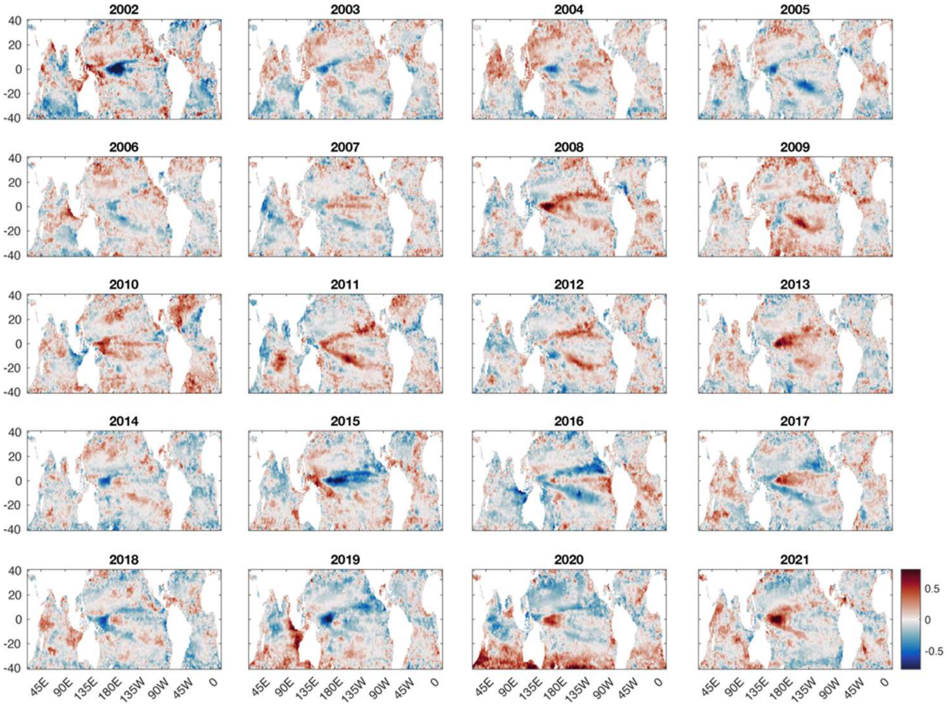

Figure S14. Global year-to-year anomalies in nutrient stress. ENSO effects are clearly visible in the annual deviations in *Θ'*. Stronger El Niño events occurred in 2003, 2005, 2015, and 2019/20, with lower *Θ'* at the edge of the equatorial Pacific upwelling zone and elevated values in the western Pacific. Stronger La Niña events included 2008, 2011/12, 2015, and 2020, with higher *Θ'* in the equatorial zone. The impact of the Indian Ocean Dipole is visible in zonal deviations between the western and eastern Indian Ocean. Strong positive IOP events included 2007 and 2019, where elevated nutrient stress appears on the western side of the basin and depressed nutrient stress on the eastern side. 2010 and 2016 represent strongly negative IOP years with opposite nutrient stress patterns.

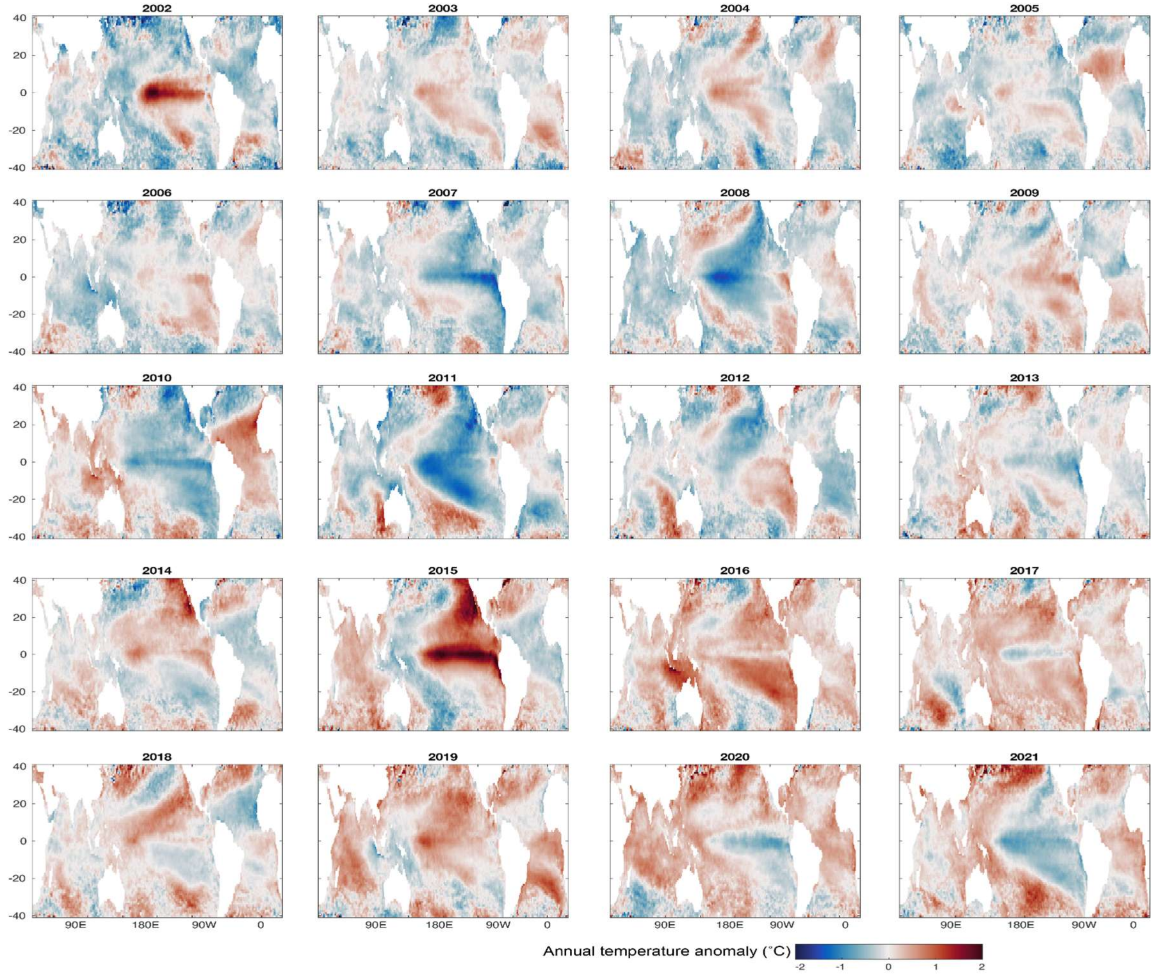

Figure S15. Yearly sea-surface temperature anomaly for 2002 – 2021. The panels capture the clear ENSO cycles in SST with strong El Niño events (e.g., 2002, 2015 and 2019) resulting in elevated temperature in the eastern Pacific and corresponding lower SST in the western Pacific Ocean. Strong La Niña events occurred in 2007/08, 2011, and 2021.

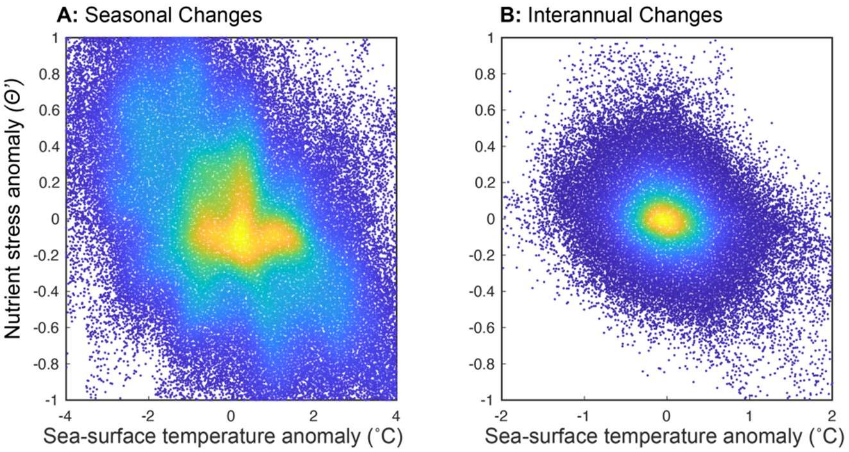

Figure S16. Correspondence between changes in temperature vs. nutrient stress. (A) Seasonal differences in temperature and *Θ'* across grid points (N = 354,240). $R_{Pearson, season}$ = -0.51, $p < 0.05$. Beyond the correlation, the panel shows that a seasonal increase in temperature clearly corresponds to a decrease in *Θ'*. **(B)** Interannual differences in temperature and *Θ'* across all grid points (N = 590,400). $R_{Pearson, annual}$ = -0.23, $p < 0.05$. Here, the overall correlation is weaker because of hemispheric differences in responses (see main manuscript) and the smaller absolute range in temperature anomalies (note the temperature scales between panels **A** and **B,** with seasonal variability being larger than interannual changes). The coloring represents the kernel sampling density

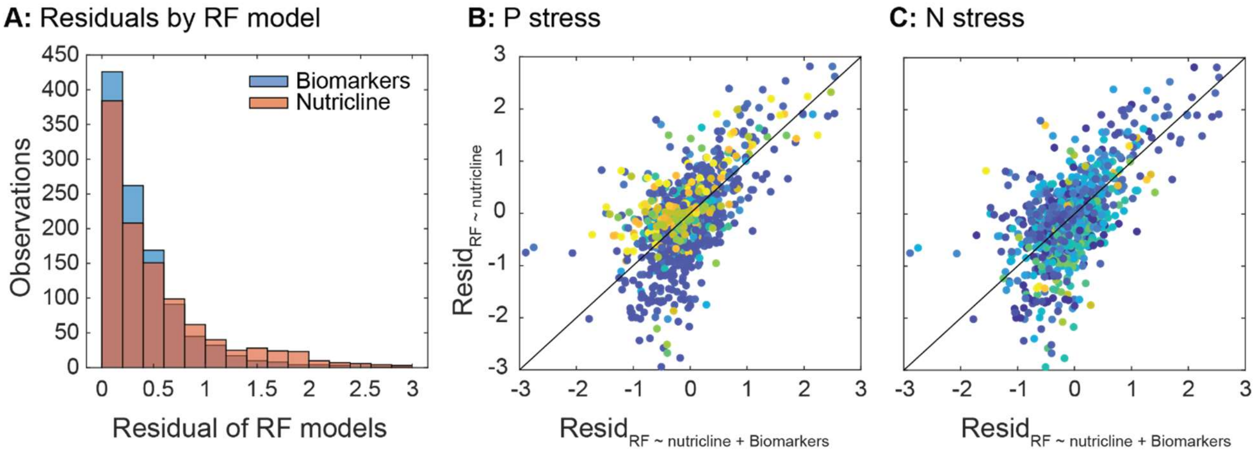

Figure S17. Comparison of Random Forest (RF) model fit residuals to observed *Θ'*. (**A**) Distribution of residuals (observed minus predicted *Θ'*) of a random forest model with only nutricline or nutricline plus biomarker information. The comparison shows a skew towards lower residuals when including biomarker information (the light brown color indicates when the RF model including biomarkers is below the RF model with only nutricline information). (**B**) Comparison of residuals between the two random forest models overlayed with high phosphate stress biomarker observations. The figure shows that Θ' is underestimated in samples with high P stress in a model with only nutricline information. (**C**) Similar to panel B, samples with high N stress display a negative skew. Thus, the incorporation of biomarker observations improved the skill of the random forest model.

Table S1: Gene Traits

Trait	Genes
**Nitrile**	*nitCD, hypAB*
**Cyanate**	*cynABDS*
**Nitrate**	*narB, napA, nirA2, narX*
**Urea**	*ureABCDEF, urtA, speB*
**Nitrite**	*nirA, focA*
**Ammonia**	*amt1, amt*

Table S2: Remote sensing and modeling data products to estimate *Θ'*

Product	Description, units	Source	Spatial	Temporal	Time period
**Chl-a**	Chlorophyll conc., mg m^{-3}	*MODIS-A	1/12º	Daily	July 2002 – 2021
**b_{bp}443**	Particul. backscat. coef., m^{-1}	MODIS-A	1/12º	Daily	July 2002 – 2021
**S_{bp}**	Backscat. spectral param	MODIS-A	1/12º	Daily	July 2002 – 2021
**K_d490**	Diffuse atten. coef., m^{-1}	MODIS-A	1/12º	Daily	July 2002 – 2021
**PAR**	Daily-integrated broadband irradi., Ein m^{-2} d^{-1}	MODIS-A	1/12º	Daily	July 2002 – 2021
**SST**	Sea-surface temperature, C	MODIS-A	1/12º	Daily	July 2002 – 2021
**MLD**	Mixed layer depth, m	@HyCOM	1/12º	Daily	July 2002 - present

*MODIS-Aqua data available from NASA OB.DAAC

@Hybrid Coordinate Model is a data assimilative, global ocean numerical model

REFERENCES

1. C. M. Moore, M. M. Mills, K. R. Arrigo, I. Berman-Frank, L. Bopp, P. W. Boyd, E. D. Galbraith, R. J. Geider, C. Guieu, S. L. Jaccard, T. D. Jickells, J. La Roche, T. M. Lenton, N. M. Mahowald, E. Maranon, I. Marinov, J. K. Moore, T. Nakatsuka, A. Oschlies, M. A. Saito, T. F. Thingstad, A. Tsuda, O. Ulloa, Processes and patterns of oceanic nutrient limitation. *Nat. Geosci.* **6**, 701–710 (2013).

2. S. D. Gerace, J. Yu, J. K. Moore, A. C. Martiny, Observed declines in upper ocean phosphate-to-nitrate availability. *Proc. Natl. Acad. Sci. U.S.A.* **122**, e2411835122 (2025).

3. G. I. Hagstrom, S. A. Levin, Marine ecosystems as complex adaptive systems: Emergent patterns, critical transitions, and public goods. *Ecosystems* **20**, 458–476 (2017).

4. M. W. Lomas, J. A. Bonachela, S. A. Levin, A. C. Martiny, Impact of ocean phytoplankton diversity on phosphate uptake. *Proc. Natl. Acad. Sci. U.S.A.* **111**, 17540–17545 (2014).

5. A. C. Martiny, G. I. Hagstrom, T. DeVries, R. T. Letscher, G. L. Britten, C. A. Garcia, E. Galbraith, D. Karl, S. A. Levin, M. W. Lomas, A. R. Moreno, D. Talmy, W. Wang, K. Matsumoto, Marine phytoplankton resilience may moderate oligotrophic ecosystem responses and biogeochemical feedbacks to climate change. *Limnol. Oceanogr.* **67**, S378–S389 (2022).

6. A. Tagliabue, L. Kwiatkowski, L. Bopp, M. Butenschön, W. Cheung, M. Lengaigne, J. Vialard, Persistent uncertainties in ocean net primary production climate change projections at regional scales raise challenges for assessing impacts on ecosystem services. *Front. Clim.* **3**, 149 (2021).

7. T. J. Browning, C. M. Moore, Global analysis of ocean phytoplankton nutrient limitation reveals high prevalence of co-limitation. *Nat. Commun.* **14**, 5014 (2023).

8. L. J. Ustick, A. A. Larkin, C. A. Garcia, N. S. Garcia, M. L. Brock, J. A. Lee, N. A. Wiseman, J. K. Moore, A. C. Martiny, Metagenomic analysis reveals global-scale patterns of ocean nutrient limitation. *Science* **372**, 287–291 (2021).

9. T. J. Browning, M. A. Saito, S. P. Garaba, X. Wang, E. P. Achterberg, C. M. Moore, A. Engel, M. R. Mcllvin, D. Moran, D. Voss, O. Zielinski, A. Tagliabue, Persistent equatorial Pacific iron limitation under ENSO forcing. *Nature* **621**, 330–335 (2023).

10. S. Jiang, F. Hashihama, H. Liu, K. Yoshitake, H. Takami, K. Hamasaki, I. Y. Ikhsani, H. Obata, H. Saito, Variations in physiology and genomic function of *Prochlorococcus* across the Eastern Indian Ocean. *J. Geophys. Res. Oceans* **128**, e2023JC019898 (2023).

11. M. J. Behrenfeld, E. Boss, D. A. Siegel, D. M. Shea, Carbon-based ocean productivity and phytoplankton physiology from space. *Global Biogeochem. Cycles* **19**, GB1006 (2005).

12. E. A. Laws, T. T. Bannister, Nutrient- and light-limited growth of *Thalassiosira fluviatilis* in continuous culture, with implications for phytoplankton growth in the ocean1. *Limnol. Oceanogr.* **25**, 457–473 (1980).

13. M. J. Behrenfeld, R. T. O'Malley, E. S. Boss, T. K. Westberry, J. R. Graff, K. H. Halsey, A. J. Milligan, D. a. Siegel, M. B. Brown, Revaluating ocean warming impacts on global phytoplankton. *Nat. Clim. Change* **6**, 323–330 (2015).

14. R. J. Geider, H. L. MacIntyre, T. M. Kana, Dynamic model of phytoplankton growth and acclimation: Responses of the balanced growth rate and the chlorophyll a:carbon ratio to light, nutrient-limitation and temperature. *Mar. Ecol. Prog. Ser.* **148**, 187–200 (1997).

15. A. A. Larkin, C. A. Garcia, N. Garcia, M. L. Brock, J. A. Lee, L. J. Ustick, L. Barbero, B. R. Carter, R. E. Sonnerup, L. D. Talley, G. A. Tarran, D. L. Volkov, A. C. Martiny, High spatial resolution global ocean metagenomes from Bio-GO-SHIP repeat hydrography transects. *Sci. Data* **8**, 107 (2021).

16. A. C. Martiny, M. W. Lomas, W. Fu, P. W. Boyd, Y.-L. L. Chen, G. A. Cutter, M. J. Ellwood, K. Furuya, F. Hashihama, J. Kanda, D. M. Karl, T. Kodama, Q. P. Li, J. Ma, T. Moutin, E. M. S. Woodward, J. K. Moore, Biogeochemical controls of surface ocean phosphate. *Sci. Adv.* **5**, eaax0341 (2019).

17. R. M. Kudela, R. C. Dugdale, Nutrient regulation of phytoplankton productivity in Monterey Bay, California. *Deep Sea Res. Part II Top. Stud. Oceanogr.* **47**, 1023–1053 (2000).

18. I. Gallego, A. Narwani, Ecology and evolution of competitive trait variation in natural phytoplankton communities under selection. *Ecol. Lett.* **25**, 2397–2409 (2022).

19. A. Herrero, A. M. Muro-Pastor, E. Flores, Nitrogen control in cyanobacteria. *J. Bacteriol.* **183**, 411–425 (2001).

20. A. C. Tolonen, J. Aach, D. Lindell, Z. I. Johnson, T. Rector, R. Steen, G. M. Church, S. W. Chisholm, Global gene expression of *Prochlorococcus* ecotypes in response to changes in nitrogen availability. *Mol. Syst. Biol.* **2**, 53 (2006).

21. H. Doré, U. Guyet, J. Leconte, G. K. Farrant, B. Alric, M. Ratin, M. Ostrowski, M. Ferrieux, L. Brillet-Guéguen, M. Hoebeke, J. Siltanen, G. Le Corguillé, E. Corre, P. Wincker, D. J. Scanlan, D. Eveillard, F. Partensky, L. Garczarek, Differential global distribution of marine picocyanobacteria gene clusters reveals distinct niche-related adaptive strategies. *ISME J.* **17**, 720–732 (2023).

22. A. C. Martiny, S. Kathuria, P. M. Berube, Widespread metabolic potential for nitrite and nitrate assimilation among *Prochlorococcus* ecotypes. *Proc. Natl. Acad. Sci. U.S.A.* **106**, 10787–10792 (2009).

23. S. E. Baer, S. Rauschenberg, C. A. Garcia, N. S. Garcia, A. C. Martiny, B. S. Twining, M. W. Lomas, Carbon and nitrogen productivity during spring in the oligotrophic Indian Ocean along the GO-SHIP IO9N transect. *Deep Sea Res. Part II Top. Stud. Oceanogr.* **161**, 81–91 (2019).

24. R. R. Malmstrom, A. Coe, G. C. Kettler, A. C. Martiny, J. Frias-Lopez, E. R. Zinser, S. W. Chisholm, Temporal dynamics of *Prochlorococcus* ecotypes in the Atlantic and Pacific oceans. *ISME J.* **4**, 1252–1264 (2010).

25. Z. Yuan, E. P. Achterberg, A. Engel, M. Dai, T. J. Browning, Switches between nitrogen limitation and nitrogen–phosphorus co-limitation in the subtropical North Atlantic Ocean. *Limnol. Oceanogr.* **69**, 1005–1013 (2024).

26. M. L. Bender, B. Jönsson, Is seasonal net community production in the South Pacific Subtropical Gyre anomalously low? *Geophys. Res. Lett.* **43**, 9757–9763 (2016).

27. S. Bonnet, C. Guieu, F. Bruyant, O. Prasil, F. Van Wambeke, P. Raimbault, T. Moutin, C. Grob, M. Y. Gorbunov, J. P. Zehr, S. M. Masquelier, L. Garczarek, H. Claustre, Nutrient limitation of primary productivity in the Southeast Pacific (BIOSOPE cruise). *Biogeosciences* **5**, 215–225 (2008).

28. J. W. Ammerman, R. R. Hood, D. A. Case, J. B. Cotner, Phosphorus deficiency in the Atlantic: An emerging paradigm in oceanography. *Eos Trans. Am. Geophys. Union* **84**, 165–170 (2003).

29. J. H. Martin, K. H. Coale, K. S. Johnson, S. E. Fitzwater, R. M. Gordon, S. J. Tanner, C. N. Hunter, V. A. Elrod, J. L. Nowicki, T. L. Coley, R. T. Barber, S. Lindley, A. J. Watson, K. Van Scoy, C. S. Law, M. I. Liddicoat, R. Ling, T. Stanton, J. Stockel, C. Collins, A. Anderson, R. Bidigare, M. Ondrusek, M. Latasa, F. J. Millero, K. Lee, W. Yao, J. Z. Zhang, G. Friederich, C. Sakamoto, F. Chavez, K. Buck, Z. Kolber, R. Greene, P. Falkowski, S. W. Chisholm, F. Hoge, R. Swift, J. Yungel, S. Turner, P. Nightingale, A. Hatton, P. Liss, N. W. Tindale, Testing the iron hypothesis in ecosystems of the equatorial Pacific Ocean. *Nature* **371**, 123–129 (1994).

30. S. A. Grodsky, J. A. Carton, C. R. McClain, Variability of upwelling and chlorophyll in the equatorial Atlantic. *Geophys. Res. Lett.* **35**, L03610 (2008).

31. N. Saji, T. Yamagata, Possible impacts of Indian Ocean Dipole mode events on global climate. *Climate Res.* **25**, 151–169 (2003).

32. K. H. Halsey, B. M. Jones, Phytoplankton strategies for photosynthetic energy allocation. *Ann. Rev. Mar. Sci.* **7**, 265–297 (2015).

33. E. Marañón, M. P. Lorenzo, P. Cermeño, B. Mouriño-Carballido, Nutrient limitation suppresses the temperature dependence of phytoplankton metabolic rates. *ISME J.* **12**, 1836–1845 (2018).

34. R. W. Eppley, Temperature and phytoplankton growth in the sea. *Fish. Bull.* **70**, 1063–1085 (1972).

35. M. J. Behrenfeld, K. M. Bisson, Neutral theory and plankton biodiversity. *Ann. Rev. Mar. Sci.* **16**, 283–305 (2024).

36. N. S. Garcia, J. A. Bonachela, A. C. Martiny, Interactions between growth-dependent changes in cell size, nutrient supply and cellular elemental stoichiometry of marine *Synechococcus*. *ISME J.* **10**, 2715–2724 (2016).

37. C. R. Benitez-Nelson, The biogeochemical cycling of phosphorus in marine systems. *Earth Sci. Rev.* **51**, 109–135 (2000).

38. P. J. Werdell, B. A. Franz, S. W. Bailey, G. C. Feldman, E. Boss, V. E. Brando, M. Dowell, T. Hirata, S. J. Lavender, Z. Lee, H. Loisel, S. Maritorena, F. Mélin, T. S. Moore, T. J. Smyth, D. Antoine, E. Devred, O. H. F. d'Andon, A. Mangin, Generalized ocean color inversion model for retrieving marine inherent optical properties. *Appl. Optics* **52**, 2019–2037 (2013).

39. M. J. Behrenfeld, L. Lorenzoni, Y. Hu, K. M. Bisson, C. A. Hostetler, P. Di Girolamo, D. Dionisi, F. Longo, S. Zoffoli, Satellite lidar measurements as a critical new global ocean climate record. *Remote Sens.* **15**, 5567 (2023).

40. A. Morel, Y. Huot, B. Gentili, P. J. Werdell, S. B. Hooker, B. A. Franz, Examining the consistency of products derived from various ocean color sensors in open ocean (Case 1) waters in the perspective of a multi-sensor approach. *Remote Sens. Environ.* **111**, 69–88 (2007).

41. E. P. Chassignet, H. E. Hurlburt, O. M. Smedstad, G. R. Halliwell, P. J. Hogan, A. J. Wallcraft, R. Baraille, R. Bleck, The HYCOM (HYbrid Coordinate Ocean Model) data assimilative system. *J. Mar. Syst.* **65**, 60–83 (2007).

42. C. D. Montegut, G. Madec, A. S. Fischer, A. Lazar, D. Iudicone, Mixed layer depth over the global ocean: An examination of profile data and a profile-based climatology. *J. Geophys. Res. Oceans* **109**, C12003 (2004).

43. R. J. Geider, H. L. MacIntyre, T. M. Kana, A dynamic model of photoadaptation in phytoplankton. *Limnol. Oceanogr.* **41**, 1–15 (1996).

44. M. J. Behrenfeld, K. H. Halsey, A. J. Milligan, Evolved physiological responses of phytoplankton to their integrated growth environment. *Philos. Trans. R. Soc. B Biol. Sci.* **363**, 2687–2703 (2008).

45. S. Menden-Deuer, E. J. Lessard, Carbon to volume relationships for dinoflagellates, diatoms, and other protist plankton. *Limnol. Oceanogr.* **45**, 569–579 (2000).

46. G. M. Silsbe, M. J. Behrenfeld, K. H. Halsey, A. J. Milligan, T. K. Westberry, The CAFE model: A net production model for global ocean phytoplankton. *Global Biogeochem. Cycles* **30**, 1756–1777 (2016).

47. R Core Team, R: A Language and Environment for Statistical Computing. (R Foundation for Statistical Computing, Vienna, 2021); https://www.R-project.org.

48. A. Liaw, M. Wiener, Classification and regression by randomForest. *R. News* **2**, 18–22 (2002).

49. A. M. Bolger, M. Lohse, B. Usadel, Trimmomatic: A flexible trimmer for Illumina sequence data. *Bioinformatics* **30**, 2114–2120 (2014).

50. B. Langmead, S. L. Salzberg, Fast gapped-read alignment with Bowtie 2. *Nat. Methods* **9**, 357–359 (2012).

51. H. Li, B. Handsaker, A. Wysoker, T. Fennell, J. Ruan, N. Homer, G. Marth, G. Abecasis, R. Durbin, The sequence alignment/map format and SAMtools. *Bioinformatics* **25**, 2078–2079 (2009).

52. A. M. Eren, Ö. C. Esen, C. Quince, J. H. Vineis, H. G. Morrison, M. L. Sogin, T. O. Delmont, Anvi'o: An advanced analysis and visualization platform for 'omics data. *PeerJ* **3**, e1319 (2015).

53. S. F. Altschul, T. L. Madden, A. A. Schaffer, J. Zhang, Z. Zhang, W. Miller, D. J. Lipman, Gapped BLAST and PSI-BLAST: A new generation of protein database search programs. *Nucleic Acids Res.* **25**, 3389–3402 (1997).

54. S. van Dongen, C. Abreu-Goodger, "Using MCL to Extract Clusters from Networks" in *Bacterial Molecular Networks: Methods and Protocols*, J. van Helden, A. Toussaint, D. Thieffry, Eds. (Springer, New York, NY, 2012), pp. 281–295; https://doi.org/10.1007/978-1-61779-361-5_15.

55. C. A. Greene, K. Thirumalai, K. A. Kearney, J. M. Delgado, W. Schwanghart, N. S. Wolfenbarger, K. M. Thyng, D. E. Gwyther, A. S. Gardner, D. D. Blankenship, The climate data toolbox for MATLAB. *Geochem. Geophys. Geosyst.* **20**, 3774–3781 (2019).

56. P. Flombaum, W.-L. Wang, F. W. Primeau, A. C. Martiny, Global picophytoplankton niche partitioning predicts overall positive response to ocean warming. *Nat. Geosci.* **13**, 116–120 (2020).

57. H. Alexander, M. Rouco, S. T. Haley, S. T. Wilson, D. M. Karl, S. T. Dyhrman, Functional group-specific traits drive phytoplankton dynamics in the oligotrophic ocean. *Proc. Natl. Acad. Sci. U.S.A.* **112**, E5972–E5979 (2015).

58. F. M. M. Morel, Kinetics of nutrient uptake and growth in phytoplankton. *J. Phycol.* **23**, 137–150 (1987).

59. M. J. Behrenfeld, A. J. Milligan, Photophysiological expressions of iron stress in phytoplankton. *Ann. Rev. Mar. Sci.* **5**, 217–246 (2013).

60. H. L. MacIntyre, T. M. Kana, T. Anning, R. J. Geider, Photoacclimation of photosynthesis irradiance response curves and photosynthetic pigments in microalgae and cyanobacteria. *J. Phycol.* **38**, 17–38 (2002).

61. J. J. Cullen, M. R. Lewis, The kinetics of algal photoadaptation in the context of vertical mixing. *J. Plankton Res.* **10**, 1039–1063 (1988).

62. K. R. Hunter-Cevera, M. G. Neubert, A. R. Solow, R. J. Olson, A. Shalapyonok, H. M. Sosik, Diel size distributions reveal seasonal growth dynamics of a coastal phytoplankter. *Proc. Natl. Acad. Sci. U.S.A.* **111**, 9852–9857 (2014).

63. A. A. Larkin, A. R. Moreno, A. J. Fagan, A. Fowlds, A. Ruiz, A. C. Martiny, Persistent El Niño driven shifts in marine cyanobacteria populations. *PLOS ONE* **15**, e0238405 (2020).

64. E. Sherman, J. K. Moore, F. Primeau, D. Tanouye, Temperature influence on phytoplankton community growth rates. *Global Biogeochem. Cycles* **30**, 550–559 (2016).
